# Supplementary material for: Functional improvement of dystrophic muscle by utrophin gene editing
Source: Mol Ther Adv. 2026 Oct 1;34(4):201857. doi: 10.1016/j.omta.2026.201857 (PMC13718585; doi:10.1016/j.omta.2026.201857)
Supplement: Supplementary file 1 [file mmc1.pdf]

OMTA, Volume *34*

Supplemental information

Functional improvement of dystrophic muscle by utrophin gene editing

Trinath Ghosh, SK Tousif Ahamed, Junchao Xu, Xuexiang Han, Istvan Tombacz, Lesley S. Chaboub, Michael J. Mitchell, Drew Weissman, and Tejvir S. Khurana

Supplemental Methods

Vector genome quantification

Vector genome copy number was quantified by SYBR Green–based quantitative PCR (qPCR) using primer pairs specific to each sgRNA cassette (Table S6). For the ΔIMTR (sg6/sg4) vector construct, primer pair F1/R was used, whereas primer pair F2/R was used for $\Delta IMTR^{+}$(sg1/sg5) vector construct detection. Genomic DNA was isolated from heart, liver, kidney, gastrocnemius, and diaphragm tissues collected from treated and control *mdx* mice and used as the qPCR template. Standard curves were generated using gel-purified PCR amplicons corresponding to each target sequence, enabling absolute quantification of vector genome copies.

In Vitro transcribed mRNA production

The plasmid encoding codon-optimized Cas9, flanked between optimized untranslated regions and containing a 101-base poly(A) tail, was supplied by GenScript (Piscataway, NJ, USA) as 1 mg/ml industrial-grade, endotoxin-free plasmid in nuclease-free water. The plasmid was linearized, purified and subjected to *in vitro* transcription using MEGAscript T7 transcription kits (Thermo Fisher Scientific, Waltham, MA, USA) and co-transcriptional capping with CleanCap (TriLink Biotechnologies, San Diego, CA, USA). Uridines were fully substituted with N1-methylpseudouridine (TriLink Biotechnologies) [1–3] mRNA was cellulose-purified to remove double stranded contaminants, as previously described.[4]

LNP formulation

LNPs were formulated by microfluidic mixing using an ionizable lipid (5D8), DOPE, cholesterol, and DMG-PEG2000 at a molar ratio of 40:10:48.5:1.5. Lipids were dissolved in ethanol and mixed with RNA diluted in 10 mM citrate buffer (pH 3.0) at an ethanol-to-aqueous flow rate ratio of 1:3. The ionizable lipid-to-RNA ratio was maintained at 10:1 (w/w). For *Utrn* gene-editing cargo formulations, Cas9 mRNA and two sgRNAs were encapsulated at a weight ratio of 2:1:1 (Cas9 mRNA:sgRNA1:sgRNA2). Following formulation, LNPs were dialyzed into 1× PBS using a 20 kDa molecular weight cut-off membrane for 2 h, sterile filtered through a 0.22 µm filter, and stored at 4 °C until use. The resulting LNPs typically exhibited a particle diameter of ~80–120 nm with RNA encapsulation efficiency exceeding 90%.

LNP and sgRNAs/SpCas9 mRNA transfection

For LNP–based delivery, LNP formulations were added directly to C2C12 cells plated in 24-well plates. For mRNA-based delivery, dual sgRNAs and SpCas9 mRNA were co-transfected using Lipofectamine™ MessengerMAX™ Transfection Reagent (#LMRNA001, Invitrogen) following the manufacturer's protocol. Cells were harvested 48 h post-transfection for genomic DNA isolation.

Supplemental Tables

Table S1. Single guide RNAs used for *in vitro* transfection.

No.	Name of sgRNA	**Sequence (5'-3')**	**gRNA/PAM Orientation**
1	sg1	mG*mC*mU* rUrArC rUrGrG rGrUrG rUrUrC rUrArC rUrGrG rUrUrU rUrArG rArGrC rUrArG rArArA rUrArG rCrArA rGrUrU rArArA rArUrA rArGrG rCrUrA rGrUrC rCrGrU rUrArU rCrArA rCrUrU rGrArA rArArA rGrUrG rGrCrA rCrCrG rArGrU rCrGrG rUrGrC mU*mU*mU* rU	GCTTACTGGGTGTTCTACTG/AGG Forward
2	sg2	mA*mG*mG* rArArU rGrUrA rArUrA rArUrG rUrArA rArGrG rUrUrU rUrArG rArGrC rUrArG rArArA rUrArG rCrArA rGrUrU rArArA rArUrA rArGrG rCrUrA rGrUrC rCrGrU rUrArU rCrArA rCrUrU rGrArA rArArA rGrUrG rGrCrA rCrCrG rArGrU rCrGrG rUrGrC mU*mU*mU* rU	AGGAATGTAATAATGTAAAG/TGG Reverse
3	sg3	mC*mA*mC* rCrArU rCrCrU rUrUrA rGrUrA rArCrC rArCrG rUrUrU rUrArG rArGrC rUrArG rArArA rUrArG rCrArA rGrUrU rArArA rArUrA rArGrG rCrUrA rGrUrC rCrGrU rUrArU rCrArA rCrUrU rGrArA rArArA rGrUrG rGrCrA rCrCrG rArGrU rCrGrG rUrGrC mU*mU*mU* rU	CACCATCCTTTAGTAACCAC/TGG Forward
4	sg4	mG*mA*mG* rUrGrU rGrUrU rCrArA rCrUrA rGrGrU rArUrG rUrUrU rUrArG rArGrC rUrArG rArArA rUrArG rCrArA rGrUrU rArArA rArUrA rArGrG rCrUrA rGrUrC rCrGrU rUrArU rCrArA rCrUrU rGrArA rArArA rGrUrG rGrCrA rCrCrG rArGrU rCrGrG rUrGrC mU*mU*mU* rU	GAGTGTGTTCAACTAGGTAT/TGG Forward
5	sg5	mA*mC*mG* rArCrA rCrCrA rArArU rCrUrA rCrArA rCrUrG rUrUrU rUrArG rArGrC rUrArG rArArA rUrArG rCrArA rGrUrU rArArA rArUrA rArGrG rCrUrA rGrUrC rCrGrU rUrArU rCrArA rCrUrU rGrArA rArArA rGrUrG rGrCrA rCrCrG rArGrU rCrGrG rUrGrC mU*mU*mU* rU	ACGACACCAAATCTACAACT/TGG Reverse
6	sg6	mC*mC*mU* rCrUrU rArUrU rUrArU rUrArC rUrCrA rGrArG rUrUrU rUrArG rArGrC rUrArG rArArA rUrArG rCrArA rGrUrU rArArA rArUrA rArGrG rCrUrA rGrUrC rCrGrU rUrArU rCrArA rCrUrU rGrArA rArArA rGrUrG rGrCrA rCrCrG rArGrU rCrGrG rUrGrC mU*mU*mU* rU	CCTCTTATTTATTACTCAGA/AGG Reverse

* 2′-O-methyl + phosphorothioate modification

Table S2. Primers used to amplify unedited wild type and deletion mutant alleles of *Utrn* 3'UTR.

Primers	Orientation	Sequence (5'-3')
P1	Forward	GTATTGCCCTCTTCTGCAAATGC
P2	Reverse	TAGATGACAGTCTGCGATTAACCAC

Table S3. *Ex-vivo* functional assessment of EDL.

	n	*mdx*	*mdx*/ΔIMTR	*mdx*/ΔIMTR+
**Twitch force (mN)**	4	25.58 ± 3.973	30.83 ± 2.901, P=0.3272	26.07 ± 3.504, P=0.9293
**Specific twitch force (mN/mm^2)**	4	31.54 ± 4.286	41.17 ± 4.196, P=0.1595	36.51 ± 3.549, P=0.4059
**Tetanic force (mN)**	4	136.4 ± 10.71	165.5 ± 17.98, P=0.2141	156.9 ± 24.84, P=0.4765
**Specific tetanic force (mN/mm^2)**	4	169.1 ± 10.42	217.6 ± 12.77, P=0.0257*	214.9 ± 14.14, P=0.04*
**ECC force drop (Contraction 1-7, %)**	3, 2, 3	19.24 ± 4	7.23 ± 1.5, P<0.01**	7.68 ± 0.87, P<0.001***
**L0 (mm)**	4	10.48 ± 0.85	10.03 ± 0.31, P=0.6372	10.25 ± 0.32, P=0.8133
**Muscle Mass (mg)**	4	8.84 ± 0.26	8.10 ± 0.81, P=0.4202	7.88 ± 1.13, P=0.4360
**CSA (mm^2)**	4	0.81 ± 0.07	0.73 ± 0.12, P=0.5576	0.76 ± 0.06, P=0.5686
**CK (U/L)**	4+	23510 ± 10510	7691 ± 2896, P=0.1971	7031 ± 1924, P=0.1741

CSA, cross sectional area; ECC, eccentric contraction; L0, muscle length; +samples lysed; two-tailed Student's t-test (*P < 0.05, **P < 0.01, ***P < 0.001)

Table S4. Deletion efficiency following AAV transduction.

		Mean ± SEM	
**Tissue**	**n**	**% of ΔIMTR**	**% of ΔIMTR+**
**Heart**	4,3	15.42 ± 4.35	16.14 ± 2.42
**Kidney**	4	12.80 ± 3.07	12.04 ± 3.43
**Liver**	4	10.87 ± 1.16	12.71 ± 2.49
**Gastrocnemius**	4	18.70 ± 3.84	16.63 ± 3.45
**Diaphragm**	4	20.13 ± 5.87	15.36 ± 3.87

Note: Heart n=4 for ΔIMTR and n=3 for ΔIMTR+

Table S5. Complementary DNA oligonucleotides for cloning and expression of sgRNA

Oligonucleotides	Sequence (5'-3')
sg1_F	ACCGCTTACTGGGTGTTCTACTG
sg1_R	AACCAGTAGAACACCCAGTAAGC
sg4_F	ACCGAGTGTGTTCAACTAGGTAT
sg4_R	AACATACCTAGTTGAACACACTC
sg5_F	ACCACGACACCAAATCTACAACT

sg5_R	AACAGTTGTAGATTTGGTGTCGT
sg6_F	ACCCCTCTTATTTATTACTCAGA
sg6_R	AACTCTGAGTAATAAATAAGAGG

Table S6. Primers used for vector genome quantification.

Primer	Orientation	Sequence (5'-3')
F1	Forward	CCGGAGTGTGTTCAACTAGGTATG
F2	Forward	CCGACGACACCAAATCTACAACTG
R	Reverse	CGGGCCATTTACCGTAAGTTATGTAAC

Table S7. Primers used to quantify deletion efficiency.

Primers	Orientation	Sequence (5'-3')
ΔP1	Forward	CAACAGAAACTCCCGGAACTCA
P2	Reverse	TAGATGACAGTCTGCGATTAACCAC
GAPDH_F	Forward	ATCACCATCTTCCAGGAGC
GAPDH_R	Reverse	CCTTCTCCATGGTGGTGAAG

Table S8. Primers used for amplicon sequencing.

Primer	Orientation	Sequence (5'-3')
sg6/4_F	Forward	TTGTATTTTTCGAGTCCTGTGTAAGTTTTGAAAGC
sg6/4_R	Reverse	TATTTAGATTTATCAGAATATAGATCATCTTATAGTTAGCTTTCATCACATACATTCAGA
sg1/5_F	Forward	GTTCTGAAAGATGCCCTTATTTTCCCATG
sg1/5_R	Reverse	ATACGGTATGTAACAGATTAGATGACAGTCTGC

Supplemental Figures

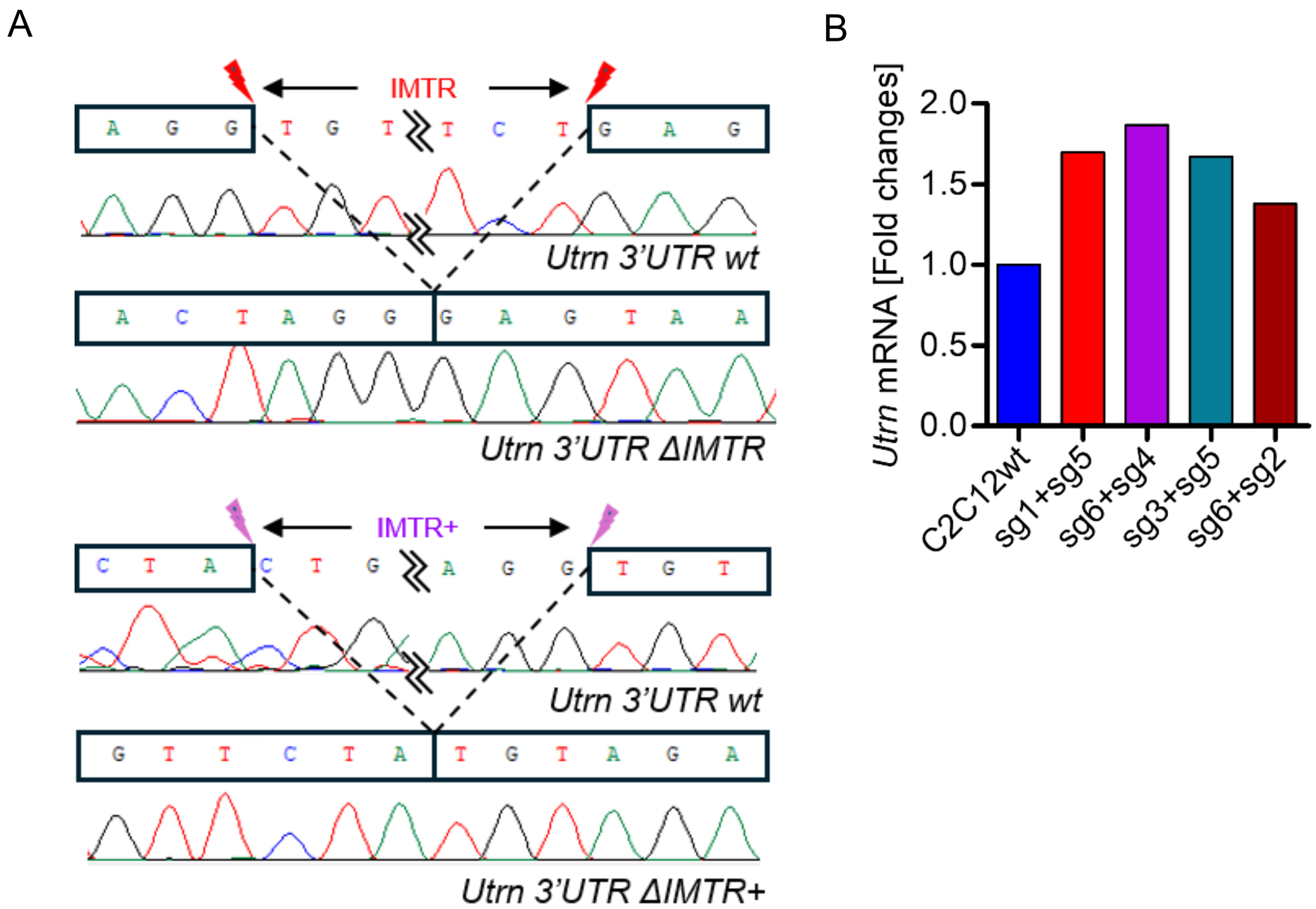

Figure S1. *In vitro* utrophin gene editing increases *Utrn* mRNA expression in *C2C12* muscle cell line.

A. Illustrative chromatograms of DNA sequence from PCR amplicons of unedited wt, ΔIMTR and ΔIMTR^{+} edited C2C12 muscle cell clonal lines, confirming appropriate deletion relative to the unedited DNA. The red and purple bolts show the position of the Cas9 editing sites.

B. TaqMan RT–qPCR analysis of *Utrn* mRNA levels in C2C12 wt and clonal *Utrn* Δ3′UTR cell lines (sg1+sg5, sg6+sg4, sg3+sg5 and sg6+sg2). All clonal *Utrn* Δ3′UTR cell lines showed increased expression compared with C2C12 wt cell line (n=3, technical replicates). Expression was normalized to *Gapdh*.

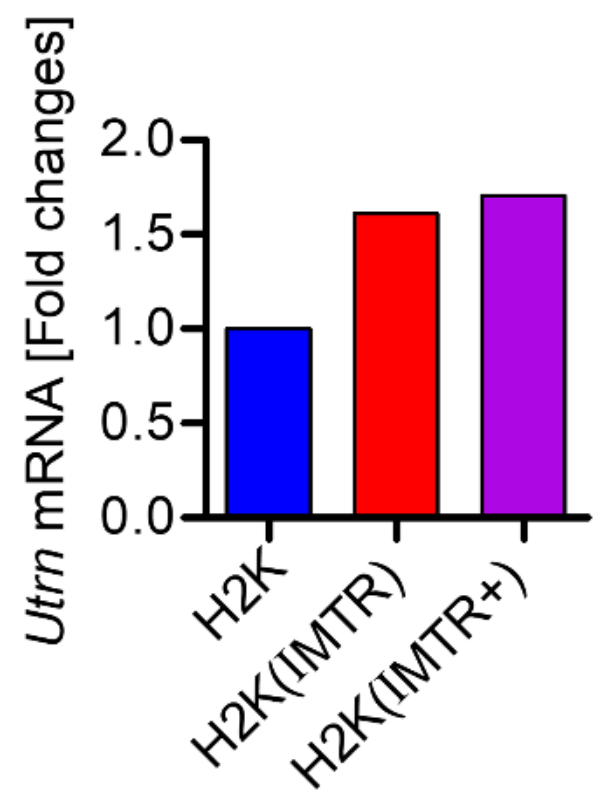

Figure S2. *In vitro* utrophin gene editing increases *Utrn* mRNA expression in MyoAAV2A-ΔIMTR/ΔIMTR$^+$ transduced differentiated H2K cells. TaqMan RT–qPCR analysis of *Utrn* mRNA levels in untreated, MyoAAV2A-ΔIMTR, and MyoAAV2A-ΔIMTR$^+$ transduced differentiated H2K cells. Both MyoAAV2A-ΔIMTR, and MyoAAV2A- ΔIMTR$^+$ transduced H2K cells showed increased expression compared with untreated (n = 3, technical replicates). Expression was normalized to *Gapdh*.

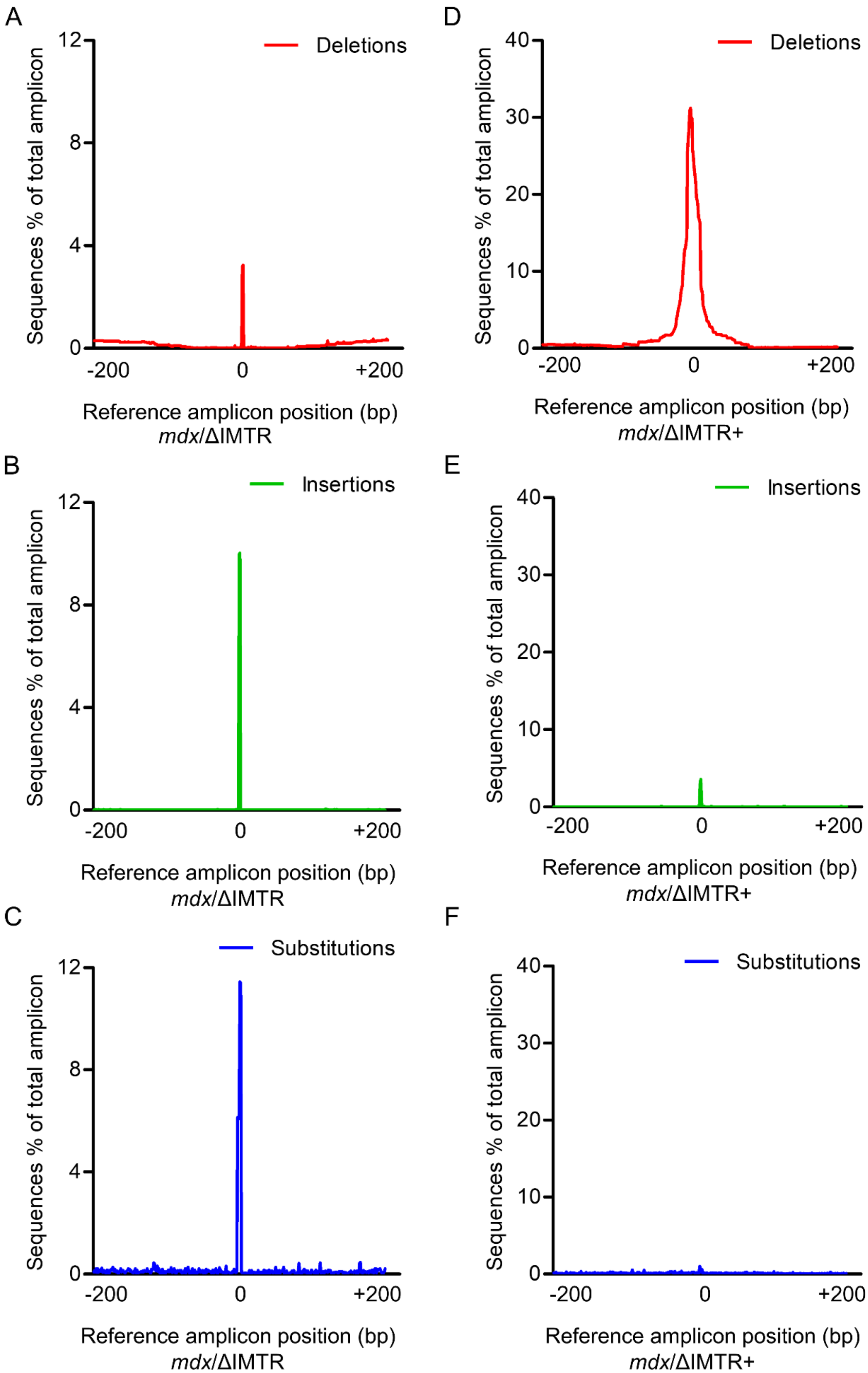
A
Deletions
Sequences % of total amplicon
12
8
4
0
-200
0
+200
Reference amplicon position (bp)
mdx/ΔIMTR
B
Insertions
Sequences % of total amplicon
12
8
4
0
-200
0
+200
Reference amplicon position (bp)
mdx/ΔIMTR
C
Substitutions
Sequences % of total amplicon
12
8
4
0
-200
0
+200
Reference amplicon position (bp)
mdx/ΔIMTR
D
Deletions
Sequences % of total amplicon
40
30
20
10
0
-200
0
+200
Reference amplicon position (bp)
mdx/ΔIMTR+
E
Insertions
Sequences % of total amplicon
40
30
20
10
0
-200
0
+200
Reference amplicon position (bp)
mdx/ΔIMTR+
F
Substitutions
Sequences % of total amplicon
40
30
20
10
0
-200
0
+200
Reference amplicon position (bp)
mdx/ΔIMTR+

G

Group		% Total mutation	bp from center
mdx/ΔIMTR	M5	57.3	±100
mdx/ΔIMTR	M6	49.8	±100
mdx/ΔIMTR	M7	53.9	±100
mdx/ΔIMTR	M8	56.5	±100
mdx/ΔIMTR^{+}	M9	91.8	±100
mdx/ΔIMTR^{+}	M10	92.8	±100
mdx/ΔIMTR^{+}	M11	92.5	±100
mdx/ΔIMTR^{+}	M12	92.7	±100

H

Editing outcome	mdx/ΔIMTR Mean ± SEM	mdx/ΔIMTR+ Mean ± SEM
Precise cut	46.05 ± 0.53	37.52 ± 0.23
Deletion	6.79 ± 0.11	31.04 ± 0.12
Insertion	8.45 ± 0.97	2.25 ± 0.03
Substitution	29.31 ± 1.03	14.10 ± 0.10
Deletion + Insertion	1.02 ± 0.09	0.21 ± 0.01
Deletion + Substitution	4.70 ± 0.25	13.42 ± 0.15
Insertion + Substitution	3.13 ± 0.19	1.10 ± 0.03
Deletion + Insertion + Substitution	0.56 ± 0.11	0.35 ± 0.02

Figure S3. Distribution and classification of sequence modifications following *in vivo Utrn* 3′UTR editing in *mdx* mice.

A–C. Representative position-specific frequencies of deletions (Red), insertions (Green), and substitutions (Blue) across the reference amplicon in the *mdx*/ΔIMTR group, generated from CRISPResso2 analysis using the expected precise deletion amplicon as the reference sequence. Mutation frequencies are plotted as the percentage of total aligned reads at each nucleotide position.

D–F. Representative position-specific frequencies of deletions (Red), insertions (Green), and substitutions (Blue) in the *mdx*/ΔIMTR^{+} group. generated from CRISPResso2 analysis using the expected precise deletion amplicon as the reference sequence. Mutation frequencies are plotted as the percentage of total aligned reads at each nucleotide position.

G. Quantitation of position-specific frequencies of mutations. Table shows the percentage of mutations that occurred +/- 100 bp from the center of the amplicon in *mdx*/ΔIMTR and *mdx*/ΔIMTR$^+$ edited mice.

H. CRISPResso2 analysis of amplicon deep sequencing data. Data regarding frequencies of precise cuts, deletions, insertions, substitutions, deletion plus insertion, deletion plus substitution, insertion plus substitution, combined deletion, insertion, and substitution events in *mdx/*ΔIMTR and *mdx*/ΔIMTR$^+$ mice. Data are presented as mean ± SEM (n = 4 mice per group).

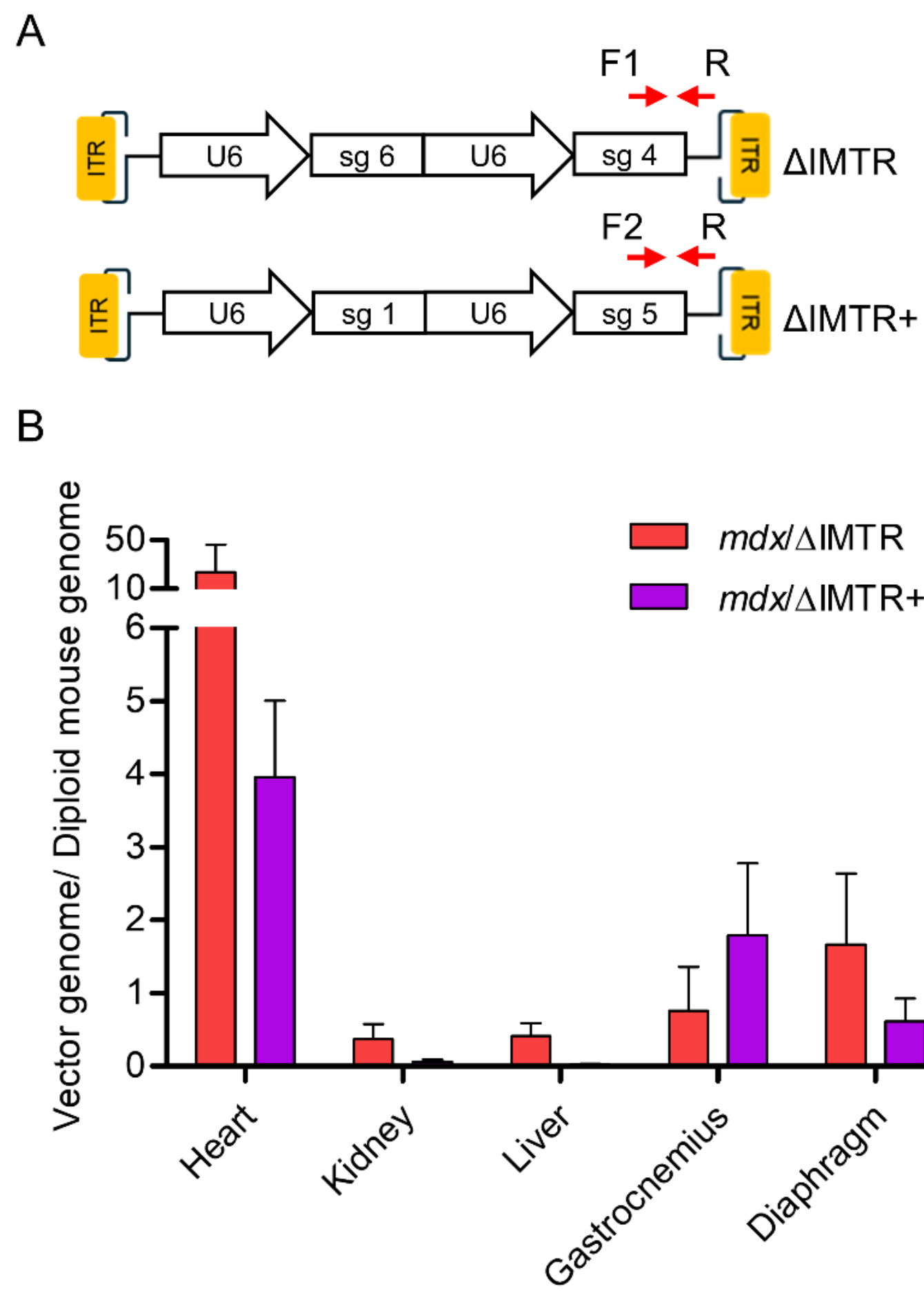

Figure S4. MyoAAV2A vector genome copy number in tissues following systemic Cas9/sgRNA delivery.

A. Schematic representation of the MyoAAV2A vector constructs used for *in vivo* genome editing, indicating primer binding sites for qPCR analysis. Primer pairs F1/R and F2/R were designed to specifically amplify the *mdx*/ΔIMTR and *mdx*/ΔIMTR$^+$ vector constructs delivering the sg6/sg4 and sg1/sg5 RNAs, respectively.

B. qPCR–based quantification of MyoAAV2A vector genome copy number of the sgRNA delivery vector, in indicated tissues (heart, kidney, liver, gastrocnemius, and diaphragm) harvested 4 weeks after intraperitoneal (IP) injection. Data are presented as mean ± SEM (n = 4 mice per group).

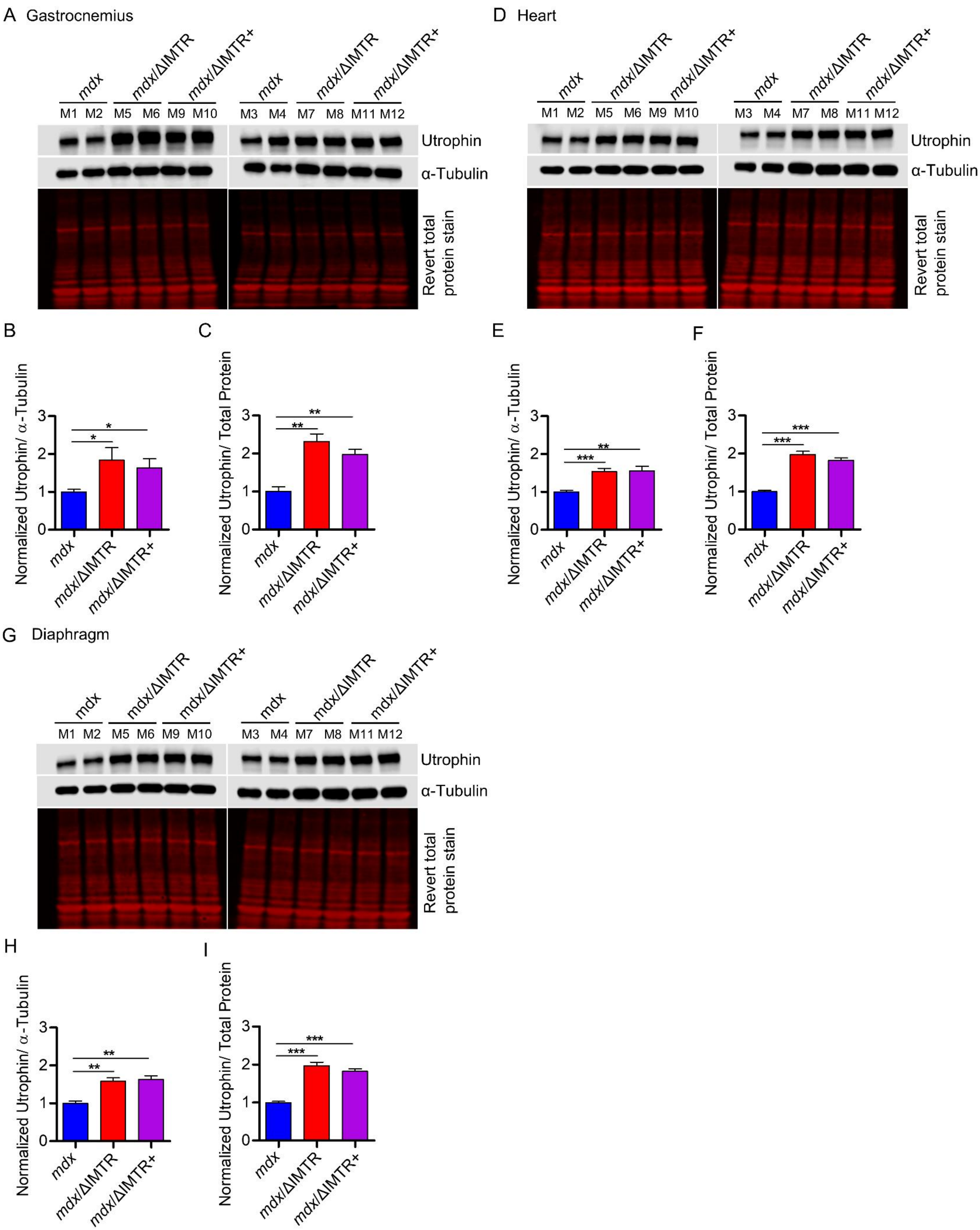
A Gastrocnemius
mdx
mdx/ΔIMTR
mdx/ΔIMTR+
M1 M2 M5 M6 M9 M10
M3 M4 M7 M8 M11 M12
Utrophin
α-Tubulin
Revert total protein stain
D Heart
B
C
E
F
Normalized Utrophin/ α-Tubulin
Normalized Utrophin/ Total Protein
G Diaphragm
H
I

Figure S5. *In vivo* utrophin gene editing increases utrophin expression in gastrocnemius, heart and diaphragm of *mdx* mice.

A. Representative Western blot showing utrophin protein expression in gastrocnemius muscle lysates from *mdx*, *mdx*/ΔIMTR, and *mdx*/ΔIMTR$^+$ mice. α-tubulin and total protein staining (Revert™700) were used for normalization controls.

B–C. Quantification of utrophin protein expression in gastrocnemius muscle normalized to α-tubulin **(B)** or total protein **(C)**. Data are presented as mean ± SEM (n = 4 mice per group). Relative to *mdx* controls, utrophin levels increased by ~84% and ~64% in muscle with α-tubulin normalization, and by ~132% and ~98% in muscle with total protein normalization, in the *mdx*/ΔIMTR and *mdx*/ΔIMTR$^+$ groups, respectively.

D. Representative Western blot showing utrophin protein expression in heart lysates from mdx, *mdx*/ΔIMTR, and *mdx*/ΔIMTR$^+$ mice. α-tubulin and total protein staining (Revert™700) were used as loading controls.

E–F. Quantification of utrophin protein expression in heart tissue normalized to α-tubulin **(E)** or total protein **(F)**. Data are presented as mean ± SEM (n = 4 mice per group). Relative to *mdx* controls, utrophin levels increased by ~54% and ~56% with α-tubulin normalization, and by ~97% and ~82% with total protein normalization, in the *mdx*/ΔIMTR and *mdx*/ΔIMTR$^+$ groups, respectively.

G. Representative Western blot showing utrophin protein expression in diaphragm lysates from *mdx*, *mdx*/ΔIMTR, and *mdx*/ΔIMTR$^+$ mice. α-tubulin and total protein staining (Revert™700) were used as loading controls.

H–I. Quantification of utrophin protein expression in diaphragm tissue normalized to α-tubulin **(H)** or total protein **(I)**. Data are presented as mean ± SEM (n = 4 mice per group). Relative to *mdx* controls, utrophin levels increased by ~58% and ~63% with α-tubulin normalization, and by ~95% and ~89% with total protein normalization, in the *mdx*/ΔIMTR and *mdx*/ΔIMTR$^+$ groups, respectively.

Statistical significance was determined using a two-tailed Student's *t*-test. P values are indicated as follows: *P < 0.05, **P < 0.01, and ***P < 0.001.

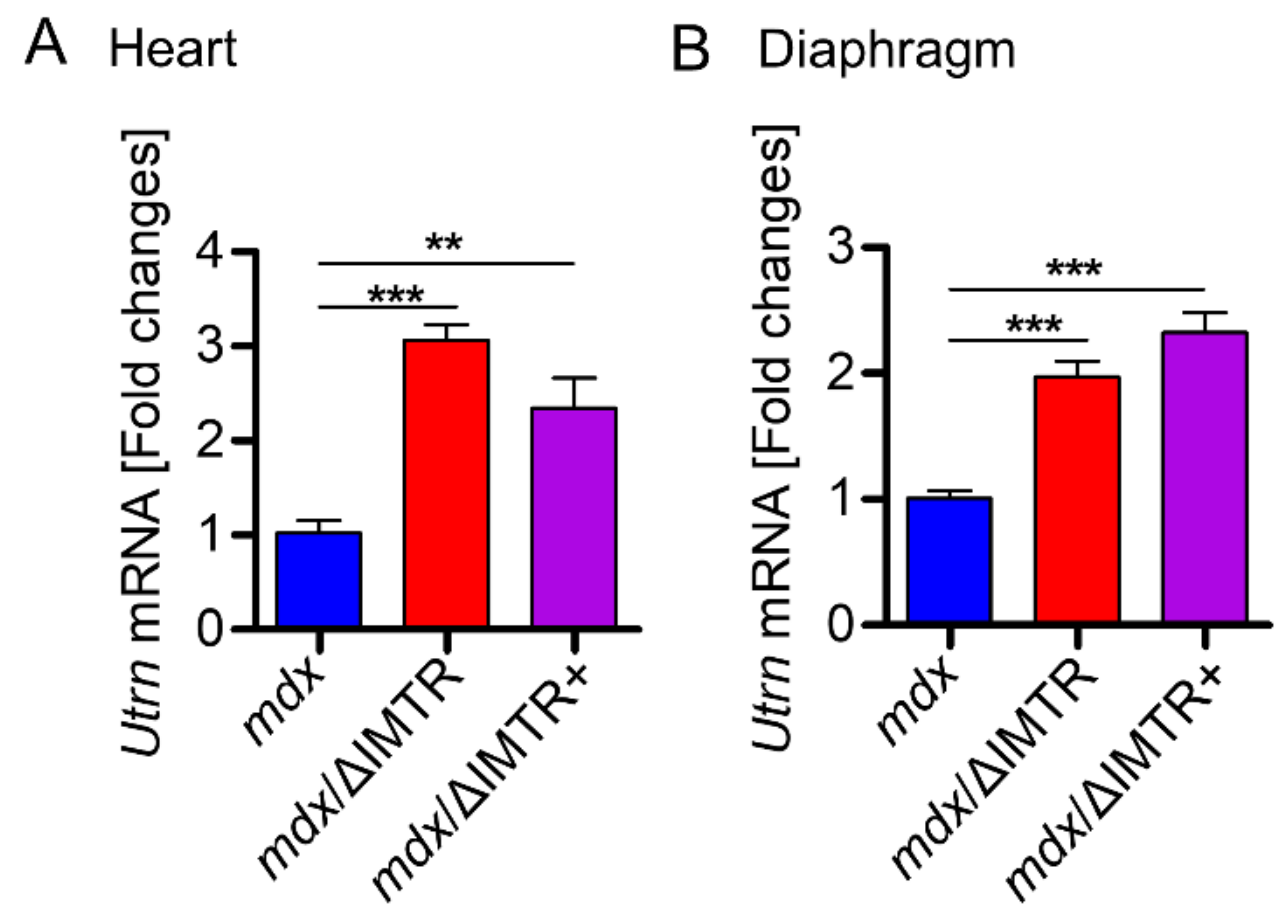

Figure S6. *In vivo* utrophin gene editing increases *Utrn* mRNA expression in *mdx* cardiac and diaphragm muscle.

A-B. TaqMan RT–qPCR analysis of *Utrn* mRNA levels in heart and diaphragm. Both *mdx*/ΔIMTR and *mdx*/ΔIMTR^{+} groups showed increased expression compared with PBS-treated *mdx* controls. Expression was normalized to *Gapdh*. Data are presented as mean ± SEM (n = 4 mice per group). Statistical significance was determined using a two-tailed Student's *t*-test (**P < 0.01, ***P < 0.001).

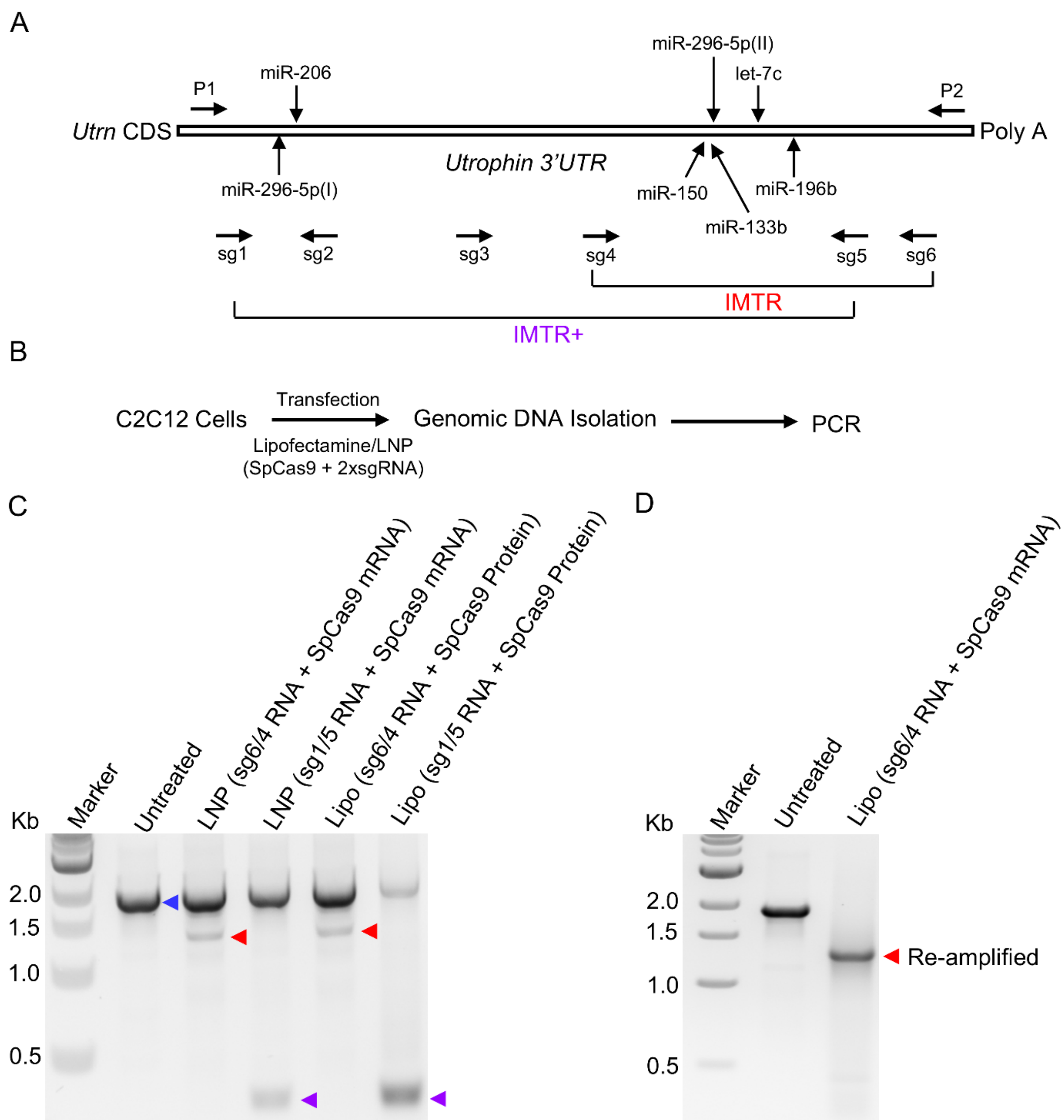

Figure S7. LNP-mediated delivery and editing of *Utrn* 3′UTR IMTR and IMTR+ in the C2C12 muscle cell line.

A. Schematic representation of the *Utrn* 3′UTR illustrating the relative positions of predicted microRNA target sites and the designed sgRNAs (sg1–sg6). sgRNA orientations are indicated by black arrows. Primer pairs (P1 and P2, red arrows) were used to amplify the targeted *Utrn* 3′UTR region.

B. Experimental workflow depicting C2C12 cell transfection using either LNP–mediated delivery of sgRNA pairs with SpCas9 mRNA or Lipofectamine-mediated delivery of sgRNA pairs with SpCas9 mRNA or protein, followed by genomic DNA isolation and PCR-based deletion screening.

C-D. Agarose gel electrophoresis of genomic PCR products from C2C12 cells transfected with different sgRNA combinations. The unedited *Utrn* 3′UTR yields an approximately 1.9 kb amplicon (blue arrow), whereas successful genome editing generates shorter deletion products corresponding to ΔIMTR (red arrows) and ΔIMTR^{+} (purple arrows).

References

1. Karikó, K., Muramatsu, H., Welsh, F.A., Ludwig, J., Kato, H., Akira, S., and Weissman, D. (2008). Incorporation of Pseudouridine Into mRNA Yields Superior Nonimmunogenic Vector With Increased Translational Capacity and Biological Stability. Mol. Ther. *16*, 1833–1840. https://doi.org/10.1038/mt.2008.200.

2. Karikó, K., Muramatsu, H., Keller, J.M., and Weissman, D. (2012). Increased Erythropoiesis in Mice Injected With Submicrogram Quantities of Pseudouridine-containing mRNA Encoding Erythropoietin. Mol. Ther. *20*, 948–953. https://doi.org/10.1038/mt.2012.7.

3. Andries, O., Mc Cafferty, S., De Smedt, S.C., Weiss, R., Sanders, N.N., and Kitada, T. (2015). N1-methylpseudouridine-incorporated mRNA outperforms pseudouridine-incorporated mRNA by providing enhanced protein expression and reduced immunogenicity in mammalian cell lines and mice. J. Controlled Release *217*, 337–344. https://doi.org/10.1016/j.jconrel.2015.08.051.

4. Baiersdörfer, M., Boros, G., Muramatsu, H., Mahiny, A., Vlatkovic, I., Sahin, U., and Karikó, K. (2019). A Facile Method for the Removal of dsRNA Contaminant from In Vitro-Transcribed mRNA. Mol. Ther. - Nucleic Acids *15*, 26–35. https://doi.org/10.1016/j.omtn.2019.02.018.
