# Supplementary material for: Functional improvement of dystrophic muscle by utrophin gene editing
Source: Mol Ther Adv. 2026 Oct 1;34(4):201857. doi: 10.1016/j.omta.2026.201857 (PMC13718585; doi:10.1016/j.omta.2026.201857)
Supplement: Supplementary file 2 [file mmc2.pdf]

Molecular Therapy
Advances
Original Article

Functional improvement of dystrophic muscle by utrophin gene editing

Trinath Ghosh,[1] SK Tousif Ahamed,[1] Junchao Xu,[2] Xuexiang Han,[2,4] Istvan Tombacz,[3] Lesley S. Chaboub,[3] Michael J. Mitchell,[2] Drew Weissman,[3] and Tejvir S. Khurana[1]

[1]Department of Physiology and Pennsylvania Muscle Institute, Perelman School of Medicine, University of Pennsylvania, Philadelphia, PA, USA; [2]Department of Bioengineering, University of Pennsylvania, Philadelphia, PA 19104, USA; [3]Division of Infectious Diseases and Penn Institute for RNA Innovation, Perelman School of Medicine, University of Pennsylvania, Philadelphia, PA 19104, USA

Utrophin upregulation is a promising therapeutic strategy for Duchenne muscular dystrophy (DMD). Utrophin expression is post-transcriptionally repressed via microRNAs binding the 3′ UTR of utrophin, particularly at the inhibitory microRNA target region (IMTR). Guide RNA pairs for editing the IMTR and a larger IMTR$^+$ region were designed and ranked by their ability to edit and increase utrophin expression in normal and dystrophic muscle cells *in vitro*. Optimized guide RNA pairs and SpCas9 were used to generate MyoAAV vectors and tested for the ability to ameliorate the dystrophic phenotype of the *mdx* mouse model of DMD, *in vivo*. Systemic delivery of MyoAAV targeting the IMTR and IMTR$^+$ using intraperitoneal injections in neonatal *mdx* mice resulted in efficient gene editing and increased utrophin expression in muscle. Increased utrophin was appropriately localized and accompanied by the appearance α-sarcoglycan at the sarcolemma, indicating dystrophin-associated glycoprotein complex recovery. Functional analyses revealed increased specific force generation and improved resistance to eccentric contractions. Histological analyses showed a reduction in the proportion of centrally nucleated fibers that are indicative of dystrophic pathology in the *mdx* mice. Together, this provides proof-of-concept for a novel therapeutic strategy that, in principle, would be applicable to all patients with DMD, independent of mutation status.

INTRODUCTION

Duchenne muscular dystrophy (DMD) is a severe X-linked neuromuscular disorder caused by mutations in the *DMD* gene that result in the absence of functional dystrophin, affecting approximately 1 in 5,000 live-born males worldwide.[1–3] Dystrophin is a 427 kDa cytoskeleton-associated protein and a core component of the dystrophin-associated glycoprotein complex (DGC), where it links the intracellular actin cytoskeleton and the extracellular matrix.[4,5] Loss of dystrophin destabilizes the DGC, rendering myofibers susceptible to damage during cycles of contraction and relaxation. This leads to repeated cycles of muscle degeneration and regeneration, chronic inflammation, and progressive muscle wasting, ultimately resulting in replacement of contractile tissue with fibrotic and adipose tissue.[6,7] Clinically, patients typically present in early childhood, lose ambulation in their teens, and suffer progressive skeletal muscle weakness accompanied by respiratory and cardiac failure, which commonly limits survival typically to the third decade of life.[8,9] Despite great progress in the basic understanding, pathophysiology and management of DMD, as well as recent FDA approvals of several drugs for DMD (cf. following text), there is currently no definitive curative therapy for DMD.[10,11]

A number of therapeutic strategies aimed at restoring dystrophin expression, including antisense splice skipping oligonucleotides,[12–14] stop-codon readthrough,[15] adeno-associated virus (AAV)-mediated delivery of an internally deleted form of dystrophin (μdystrophin),[16] and gene editing to remove mutated exons and force expression of an internally truncated dystrophin[17–21] are in various stages of preclinical development and clinical use. Because dystrophin expression is absent or severely reduced in DMD, all dystrophin-based approaches face a fundamental limitation since patients' immune system may recognize newly expressed dystrophin as foreign. Indeed, T cell-mediated immune response has been noted even at a low dose of 2×10^{10} vg/kg using AAV μdystrophin.[22] Additionally, currently FDA approved therapies designed to restore dystrophin are applicable only to those patients with DMD bearing specific types of mutation and/or clinical status. This is a fundamental constraint, given the wide range of causative mutations noted in patients with DMD[23] and underscores the need for developing mutation-independent therapies for DMD.

An alternative therapeutic strategy that circumvents many limitations of dystrophin restoration is upregulation of utrophin, the

Received 6 April 2026; accepted 30 September 2026;
https://doi.org/10.1016/j.omta.2026.201857.

[4]Present address: Institute of Biochemistry and Cell Biology, Shanghai Institutes for Biological Sciences, Chinese Academy of Sciences, No. 227 Yuanmei Road, Minhang District, Shanghai 20031, China

Correspondence: Tejvir S. Khurana, Department of Physiology and Pennsylvania Muscle Institute, Perelman School of Medicine, University of Pennsylvania, Philadelphia, PA, USA.
E-mail: tsk@pennmedicine.upenn.edu

© 2026 The Authors.
Published by Elsevier Inc. on behalf of The American Society of Gene and Cell Therapy.
This is an open access article under the CC BY license (http://creativecommons.org/licenses/by/4.0/).

autosomal paralog of dystrophin that shares extensive structural and functional homology with dystrophin.[24–26] Utrophin is expressed continuously in DMD muscle, albeit at much reduced levels compared to its levels in the fetus,[25,27] while dystrophin is severely reduced or absent. Importantly, utrophin, when overexpressed, can functionally substitute for the missing dystrophin[28,29] and ameliorate dystrophin-deficient muscle pathophysiology without eliciting an immune response.[30] Utrophin expression is known to be regulated at both the transcriptional and post-transcriptional levels, based on the mismatch in transcript and protein levels seen in some muscle groups[31] and specific locales in the brain.[32] Several strategies for activating the utrophin promoter by small molecules[33–35] as well as artificial transcription factors[36,37] have been described. Unfortunately, targeting the promoter has not been successful therapeutically. The promoter is devoid of motifs such as the CCAAT box that are typically associated with highly inducible genes, and only modest levels of upregulation have been achievable using small molecules (e.g., SMT C1100/ezutromid) targeting the promoter in clinical studies.[38] We and others have demonstrated that utrophin expression is also subject to significant post-transcriptional repression by microRNAs that bind cognate sites in the *Utrn* 3′ untranslated region (3′ UTR).[39,40] Targeted inhibition of individual microRNA interactions, by blockade of the let-7c binding site using site-blocking oligonucleotides (SBOs)[41] as well as using microRNA sponges or decoys,[42,43] has been shown to result in increased utrophin expression and functional improvement in the *mdx* mouse model providing evidence for post-transcriptional de-repression as a therapeutic strategy. These approaches require repeated administration constraining their translational potential. The advent of gene editing offers an opportunity to overcome these limitations by permanently removing inhibitory regulatory elements from the endogenous *Utrn* 3′ UTR. Previously, we and others have demonstrated the utility of gene editing by targeting the utrophin gene[44,45] in DMD myoblasts, DMD iPSC lines *in vitro* and in *mdx* mice *in vivo*,[46] providing the basic rationale for further translational studies. Here, we describe developing and using a MyoAAV[47] delivery-based utrophin gene-editing strategy for achieving utrophin upregulation *in vivo* (Figure 1). Using MyoAAV-based utrophin gene-editing, we demonstrate that *Utrn* 3′ UTR editing leads to utrophin upregulation, improved muscle function, and amelioration of dystrophic muscle histopathology in *mdx* mice.

RESULTS

Optimization of utrophin expression through CRISPR-Cas9-mediated deletion of inhibitory elements within the *Utrn* 3′ UTR

We have previously defined the inhibitory microRNA target region (IMTR) within the *Utrn* 3′ UTR where several microRNA binding sites are clustered.[39,44] We and others have also demonstrated the importance of this region for regulating utrophin expression by demonstrating that CRISPR-Cas9-mediated deletion of the *UTRN* IMTR resulted in an increase in utrophin protein expression in DMD-derived cell lines *in vitro*.[44,45] Computational predictions indicate that inhibitory microRNA-binding sites extend across a broader region of the *Utrn* 3′ UTR,[39] suggesting that removal of a larger post-transcriptional inhibitory region may increase utrophin expression as well (Figure 2A). Hence, aided by bioinformatic algorithms, we designed six single-guide RNAs[48] (sgRNAs; sg1–sg6; Table S1) targeting distinct regions across the *Utrn* 3′ UTR. To identify optimal sgRNA combinations for efficient deletion, pairs of sgRNAs were co-transfected with recombinant SpCas9 nuclease into C2C12 myoblasts (Figure 2B). Genomic PCR analysis using primers flanking the targeted region (Table S2) revealed deletions for multiple sgRNA combinations, yielding distinct amplicons including sg1+sg5 (331 bp), sg3+sg5 (847 bp), sg6+sg4 (1,312 bp), and sg6+sg2 (447 bp), compared with the 1,909 bp wild-type product (Figure 2C).

To determine the ability of various sgRNA-mediated deletions to increase utrophin expression, we generated clonal *Utrn* Δ3′ UTR C2C12 cells, using limiting dilutions. Individual clones were screened by PCR (Figure 2D), and deletions were confirmed by Sanger DNA sequencing of gel-purified amplicons, as illustrated by chromatograms, from clonal C2C12 cell lines (Figure S1). Western blot analysis demonstrated increased utrophin expression in multiple deletion clones, including sg1+sg5 (∼20%), sg6+sg4 (∼39%), and sg3+sg5 (∼21%) relative to wild-type C2C12 cells (Figures 2E and 2F). Increases in *Utrn* mRNA level were also observed in all the deletion clones (Figure S1). Based on location and ability to increase utrophin protein expression *in vitro*, we selected the sg6+sg4 pair and the sg1+sg5 pair as they would result in the ΔIMTR and ΔIMTR$^+$ deletions in the *Utrn* gene.

Validation of MyoAAV2A gene editing vectors encoding SpCas9 and *Utrn*-targeting sgRNAs in dystrophic muscle cells, *in vitro*

We generated MyoAAV2A vectors encoding SpCas9, sg6/sg4 and sg1/sg5 for targeting the *Utrn* 3′ UTR IMTR and IMTR$^+$ respectively (Figure 3A). To assess editing efficiency *in vitro*, differentiated dystrophic H2K (dystrophin deficient) mouse muscle myotubes[49] were transduced with MyoAAV2A-sg6/sg4 or MyoAAV2A-sg1/sg5 in combination with MyoAAV2A-SpCas9 at a 1:1 vector genome ratio (total dose: 5×10^8 vg per well in a 24-well plate). Genomic PCR analysis 48 h post-transduction of cells confirmed efficient deletion of the targeted *Utrn* 3′ UTR regions, yielding the expected ΔIMTR (∼1.3 kb) and ΔIMTR$^+$ (∼331 bp) products (Figure 3B). Western blot analysis revealed significant increases in utrophin levels in both ΔIMTR (∼59%) and ΔIMTR$^+$ (∼51%) cells compared with untreated H2K cells (Figures 3C and 3D). Increases in *Utrn* mRNA level were observed in transduced differentiated H2K cells (Figure S2).

Neonatal intraperitoneal delivery of MyoAAV2A-SpCas9/sgRNAs improves muscle function in *mdx* mice

To evaluate therapeutic efficacy *in vivo*, neonatal (postnatal day 7) *mdx* mice were administered MyoAAV2A-sg6/sg4 or MyoAAV2A-sg1/sg5 together with MyoAAV2A-SpCas9 via intraperitoneal injection at a 1:1 vector genome ratio (total dose: 1.5×10^{14} vg/kg), with PBS-treated *mdx* mice serving as controls (Figure 4A). Four weeks post-injection, mice were euthanized and analyzed (for details, see Table S3). *Ex vivo* functional analysis of extensor digitorum longus (EDL) muscles demonstrated a significant

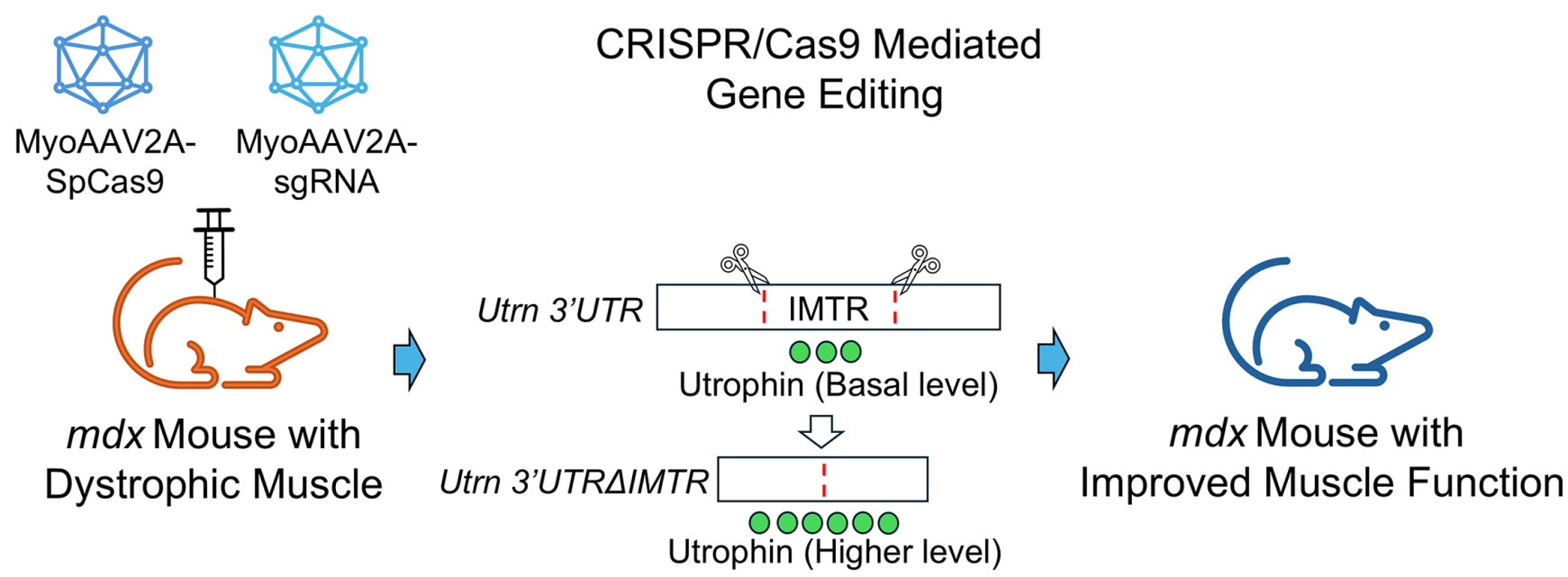

Figure 1. MyoAAV2A-mediated gene editing for utrophin upregulation in *mdx* mice

The schematic illustrates our strategy of sgRNA-guided CRISPR-Cas9-mediated deletion of the IMTR from the *Utrn* 3′ UTR by utilizing MyoAAV2A as the delivery vectors. Deletion of the IMTR/IMTR$^+$ regions would relieve the post-transcriptional repression of *Utrn*, resulting in higher levels of utrophin expression, stabilization of the dystrophin-associated glycoprotein complex, improved muscle function, and amelioration of muscle pathology in the *mdx* mouse model of DMD.

increase in isometric specific tetanic force in both *mdx*/ΔIMTR (∼28.4%) and *mdx*/ΔIMTR$^+$ (∼26.6%) mice compared with PBS-treated *mdx* controls (Figure 4B). Furthermore, both treatment groups exhibited improved resistance to post-eccentric contraction (ECC) force drop by generating significantly greater force following a series of ECCs relative to controls (Figure 4C).

Characterization of MyoAAV2A-SpCas9/sgRNAs-mediated *Utrn* gene editing, *in vivo*

To determine the efficiency of gene-editing, we isolated genomic DNA from the gastrocnemius muscle of ΔIMTR and ΔIMTR$^+$ *mdx* mice and used quantitative CRISPR PCR (QC-PCR) to determine the % reduction of the wild-type allele in edited mice.[50] This analysis demonstrated excision of the IMTR and IMTR$^+$ *in vivo*, with deletion efficiencies of ∼19% and ∼17% in the ΔIMTR and ΔIMTR$^+$ groups, respectively (Figures 5A and 5B). Comparable deletion efficiencies were also observed in the heart (ΔIMTR, ∼15%; ΔIMTR$^+$, ∼16%) and the diaphragm (ΔIMTR, ∼20%; ΔIMTR$^+$, ∼15%), indicating genome editing across multiple muscle tissues *in vivo*. Editing was also noted in the liver (ΔIMTR, ∼10.9%; ΔIMTR$^+$, ∼12.7%) and the kidney (ΔIMTR, ∼12.8%; ΔIMTR$^+$, ∼12%) (Table S4). To further characterize the mutation caused by SpCas9/sgRNAs-mediated *Utrn* gene editing *in vivo*, targeted amplicon deep sequencing was performed using the expected precise deletion amplicon as the alignment reference. In the ΔIMTR group, ∼46% of edited alleles corresponded to the intended precise deletion, whereas the remaining ∼54% contained additional sequence modifications. Similarly, in the ΔIMTR$^+$ group, ∼38% of edited alleles represented precise deletions, while ∼62% harbored modified deletions (Figure 5C). Analysis of the modified alleles revealed a heterogeneous spectrum of repair outcomes including deletions, insertions, substitutions, and complex editing events comprising multiple mutation types (Figure 5D; Figure S3). Additionally, quantitative PCR (qPCR) analysis of vector copy number of the sgRNA delivery vector revealed preferential MyoAAV2A transduction of skeletal and cardiac muscle with minimal vector presence in liver and kidney (Figure S4).

Systemic MyoAAV2A-CRISPR-mediated *Utrn* 3′ UTR editing increases sarcolemmal utrophin and stabilizes the dystrophin-associated glycoprotein complex

Western blot analysis of gastrocnemius muscle lysates revealed robust increases in utrophin protein expression in both *mdx*/ΔIMTR (∼98%) and *mdx*/ΔIMTR$^+$ (∼62%) mice relative to PBS-treated *mdx* controls (Figures 6A and 6B). Consistent with these findings, TaqMan-based qPCR analysis demonstrated significant upregulation of *Utrn* mRNA levels in treated muscles (Figure 6C), indicating that deletion of the inhibitory IMTR and IMTR$^+$ of the 3′ UTR increased both transcript abundance and protein expression. The extent of increase in utrophin expression was independently validated using a total protein-based normalization method and yielded similar results. Additionally, utrophin upregulation was noted at both protein and mRNA level in the diaphragm and heart (Figures S5 and S6).

Loss of dystrophin in dystrophic muscle destabilizes the DGC and leads to the absence of sarcolemmal labeling of DGC constituents such as α-sarcoglycan. Conversely, appearance of α-sarcoglycan[51] at the sarcolemma suggests restoration and stability of the DGC in utrophin (or dystrophin)-based therapeutic strategies. To assess the downstream impact of utrophin upregulation on DGC stability, α-sarcoglycan expression was analyzed. Western blot analysis revealed α-sarcoglycan protein levels were increased in *mdx*/ΔIMTR mice compared to PBS-treated *mdx* controls (Figures 6D and 6E). Immunofluorescent staining of tibialis anterior (TA) muscle sections demonstrated increased utrophin in both *mdx*/ΔIMTR and *mdx*/ΔIMTR$^+$ groups and was appropriately localized at the sarcolemma (Figure 7A). Co-labeling with α-bungarotoxin indicated that utrophin upregulation extended beyond neuromuscular junction regions, consistent with broad sarcolemmal restoration. Labeling for α-sarcoglycan, performed in parallel, demonstrated appearance of appropriately localized protein in both *mdx*/ΔIMTR and *mdx*/ΔIMTR$^+$ groups compared to PBS-treated *mdx* controls (Figure 7B).

MyoAAV2A-Cas9/sgRNA-mediated utrophin editing improves dystrophic muscle pathology

Histological examination of TA muscles by hematoxylin and eosin staining revealed a marked reduction in centrally nucleated fibers (CNFs), a pathological hallmark of prior regeneration in dystrophic muscle. Quantitative analysis demonstrated that both *mdx*/ΔIMTR and *mdx*/ΔIMTR$^+$ mice exhibited a significantly fewer CNFs (∼18% and ∼14%, respectively) compared to PBS-treated *mdx* controls (∼56%) (Figures 8A and 8B). This reduction in CNF proportion indicates improvement of dystrophic pathology following the MyoAAV2A-Cas9/sgRNA-mediated editing of the 3′ UTR of the

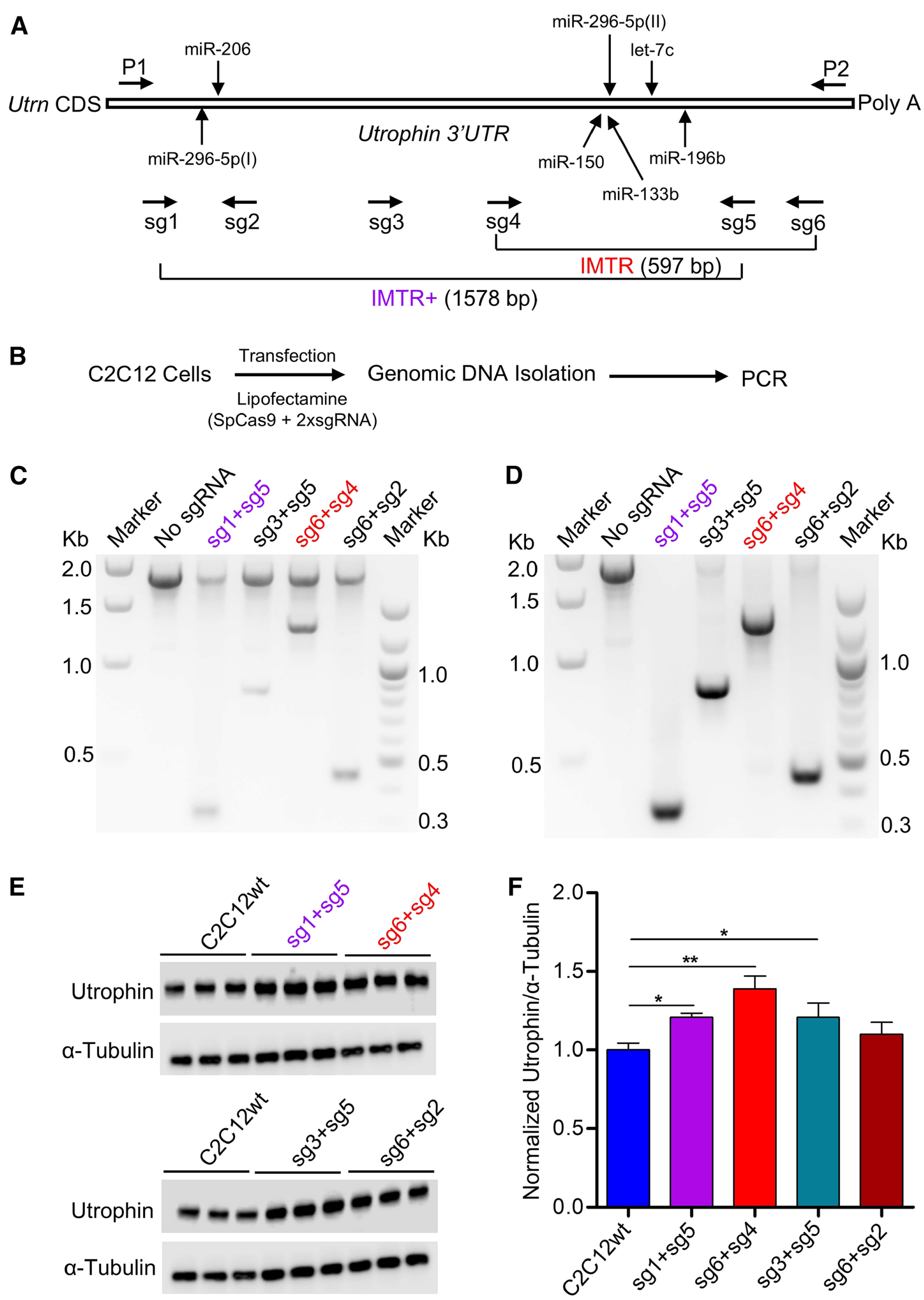

Figure 2. Design and optimization of sgRNAs targeting the *Utrn* 3′ UTR

(A) Schematic representation of the mouse *Utrn* 3′ UTR showing the relative positions of predicted microRNA target sites (miR-296-5p, miR-206, miR-133b, miR-let-7c, miR-150, and miR-196b clustered in the IMTR^{+}).[39] Relative positions of the single guide RNAs (sgRNAS; sg1–sg6; Table S1) that were designed to edit *Utrn* 3′ UTR are indicated by black arrows. Relative position of primer pairs (P1-forward and P2-reverse) that were designed to amplify the *Utrn* 3′ UTR region are indicated by arrows. sgRNAs for editing the IMTR (sgRNA6+4) are shown in red and for editing the IMTR^{+} (sgRNA1+5) in purple here and throughout. (B) Workflow for sgRNA optimization. Cultured C2C12 mouse myoblasts were transfected with selected sgRNA pairs together with recombinant SpCas9 nuclease. Genomic DNA was isolated 48 h post-transfection, followed by PCR amplification using P1 and P2 primer set and analyzed for editing by DNA agarose gel electrophoresis. (C) Representative DNA agarose gel showing PCR products from genomic DNA isolated from C2C12 cells transfected with different sgRNA combinations (sgRNA1+5, sgRNA3+5, sgRNA6+4, and sgRNA6+2). The unedited *Utrn* 3′ UTR yields a ∼1.9 kb amplicon, whereas CRISPR-Cas9-mediated deletions generate smaller products (∼330–1,300 bp) as shown. (D) Representative DNA agarose gel showing appropriately sized PCR products obtained by genotyping *Utrn*-edited C2C12 clonal cell lines that were cloned by serial dilution after transfection with sgRNA pairs and Cas9. PCR analysis and sequencing confirmed successful deletion of the targeted *Utrn* 3′ UTR region in clonal cell lines. (E) Representative western blot showing utrophin expression in wild-type (C2C12wt) and *Utrn* Δ3′ UTR C2C12 clones with different sets of sgRNAs (sgRNA1+5, sgRNA3+5, sgRNA6+4, and sgRNA6+2) as indicated; α-tubulin serves as a loading control. (F) Quantification of increased utrophin protein levels in edited cell lines. Utrophin expression data normalized to α-tubulin are presented as mean ± SEM (n = 3 independent experiments). Statistical significance was assessed using a two-tailed Student's t test (*$p < 0.05$, **$p < 0.01$).

utrophin gene and subsequent increase in utrophin protein expression.

DISCUSSION

Gene-editing technologies are increasingly being applied to develop therapeutic strategies for DMD. Here, we demonstrate that CRISPR-Cas9-mediated deletion of the IMTR or IMTR^{+} within the *Utrn* 3′ UTR using viral delivery increases endogenous utrophin expression and ameliorates dystrophic pathology in *mdx* mice. By combining optimized dual-guide RNA designs to delete microRNA binding sites in the 3′ UTR (Figures 2E and 2F) with the muscle-tropic MyoAAV delivery platform[47] (Figure 3A), we achieved increased utrophin expression in differentiated dystrophic H2K myotubes *in vitro* (Figures 3C and 3D). Systemic delivery of MyoAAV2A-Cas9/sgRNAs to neonatal *mdx* mice led to gene editing of the IMTR and IMTR^{+} in the 3′ UTR of *Utrn* in muscle and led to significant increases in utrophin mRNA and protein levels *in vivo* (Figure 6; Figures S5 and S6). The degree of utrophin upregulation was sufficient to help stabilize the DGC, as evidenced by enhanced sarcolemmal localization of utrophin, and increased α-sarcoglycan protein levels (Figures 7A and 7B). These molecular changes translated into functional improvements (Table S3), including increased specific force generation (Figure 4B), reduced susceptibility to eccentric contraction-induced injury (Figure 4C), and attenuation of

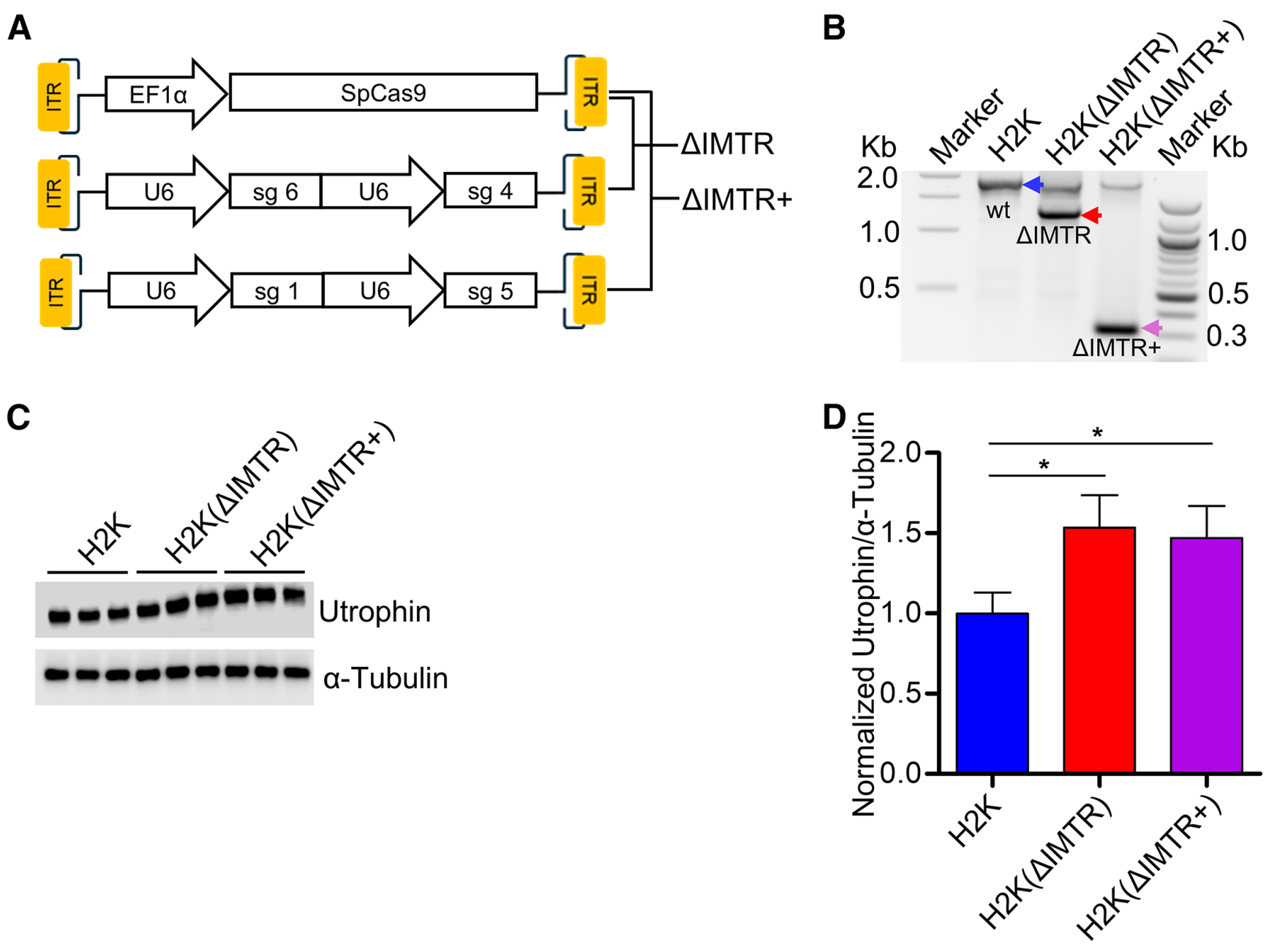

Figure 3. Design and validation of MyoAAV2A Cas9/sgRNA-mediated utrophin upregulation in dystrophic muscle cells *in vitro*

(A) Schematic of MyoAAV2A vector combinations designed to be used in tandem to generate ΔIMTR and ΔIMTR$^+$ deletions. The editing cargoes are shown flanked by inverted terminal repeats (ITRs) that enable AAV replication and packaging. The MyoAAV2A-SpCas9 was combined either with MyoAAV2A-U6-sg6/sg4 (to generate ΔIMTR) deletions or with MyoAAV2A-U6-sg1/sg5 (to generate ΔIMTR$^+$) deletions. Codon-optimized SpCas9 was driven using the EF1α promoter, and sgRNAs were driven using U6 promoters. (B) Representative DNA agarose gels showing PCR products from genomic DNA isolated from differentiated H2K muscle cells 48 h post-transduction with MyoAAVs and controls. The unedited *Utrn* 3′ UTR yields an ∼1.9 kb amplicon (blue arrowheads), whereas ΔIMTR and ΔIMTR$^+$ deletions generate appropriately sized shorter fragments (∼1.3 kb and ∼330 bp, respectively) marked with red and purple arrowheads, respectively. (C) Representative western blot showing utrophin expression in untreated, MyoAAV2A-ΔIMTR, and MyoAAV2A-ΔIMTR$^+$ transduced differentiated H2K cells; α-tubulin was used as a loading control. (D) Densitometric quantification of increased utrophin protein level in MyoAAV edited cells, normalized to α-tubulin. Data are presented as mean ± SEM (n = 3; independent experiments). Statistical significance was assessed using a two-tailed Student's t test (*$p < 0.05$).

dystrophic histopathology, as reflected by a marked reduction in CNFs (Figures 8A and 8B). Together these data provide *in vivo* proof-of concept for utrophin gene editing as a therapeutic strategy for DMD.

Previous studies have described targeting and editing dystrophin using a variety of means including CRISPR-Cas9, base/prime editors, and transcriptional activators. Although these strategies have shown promising efficacy in preclinical models,[17–19,52–56] they are inherently limited by mutation specificity, with not all *DMD* mutations (e.g., deletions containing critical functional domains) being amenable to this strategy. These strategies also require mutation-specific guide RNAs to be used since the disease can be caused by a wide range of mutations (https://www.dmd.nl/database.html). Additionally, all dystrophin-based approaches face limitations due to immunity against the newly expressed dystrophin, since patients are dystrophin-naive.[22] In contrast, dystrophin-independent gene editing strategies, such as targeting utrophin,[44,57,58] circumvent some of the limitations of the dystrophin-based gene-editing approaches. Since utrophin is expressed prior to birth, patients with DMD are not utrophin-naive[25] and hence, this strategy is predicted to have a favorable immunogenic risk profile.

Our approach selectively increases endogenous utrophin by deleting microRNA-binding sites in the *Utrn* 3′ UTR without disrupting other microRNA targets in muscle, as would be the case while using microRNA decoys. Both the *mdx*/ΔIMTR and *mdx*/ΔIMTR$^+$ mice had similar levels of utrophin upregulation and attenuation of pathology as noted in our previous *mdx in vivo* studies using 2′-O-methyl phosphorothioate let-7 SBO,[41] the "S56" let-7 morpholino SBO,[59] as well as in a recent AAV-mediated gene editing study[46] targeting the utrophin let-7 binding site[39] contained in the IMTR.[44] Greater utrophin protein upregulation was noted *in vivo* compared to the editing efficiency. This may be due to a reduction of utrophin turnover in the edited dystrophic muscle and is consistent with similar findings reported with dystrophin gene editing,[60] These studies, along with studies on KSRP and miR-206 interaction[40] and *in vitro* variant mapping data,[46] suggest the existence of motifs that may either repress or de-repress utrophin expression, depending on their location in the *Utrn* 3′ UTR, and offer an explanation for the primacy of repressive miRNAs exemplified by let-7c, binding the IMTR region compared to other locales. Deep amplicon sequencing (Figure 5) of the *in vivo* editing in muscle was consistent with the degree of Cas9-nuclease-associated imprecise editing or indels reported in the literature.[61] However, by targeting regions that lie strictly within the 3′ UTR, our strategy mitigates the potential risk of targeting a coding region, where indels may have greater consequences. The use of newer gene-editing approaches (e.g., base/prime editing) may additionally improve specificity, safety, and efficiency. We are currently developing these approaches for the utrophin gene. Additionally, utrophin upregulation-based strategies are predicted to be mutation agnostic and applicable to all patients with DMD.

Immune reactions to viral vectors are being increasingly recognized as a major cause of immunogenicity and toxicity in AAV-based gene therapy.[62–65] Our strategy of utilizing intraperitoneal delivery using

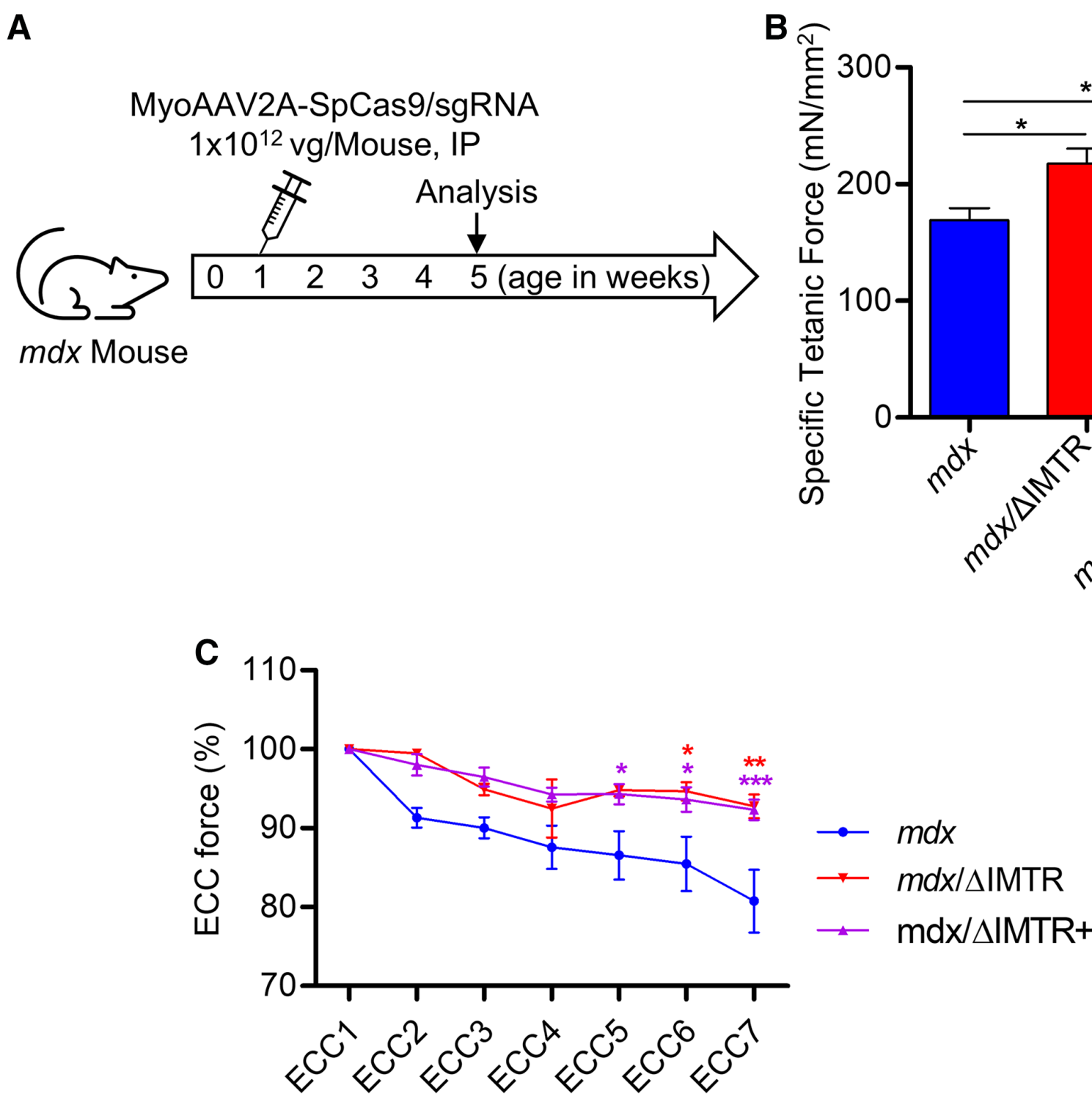

Figure 4. *In vivo* utrophin gene editing improves muscle function in *mdx* mice

(A) Neonatal (1 week old) *mdx* mice were treated with PBS (control) or MyoAAV2A-Cas9/sgRNA (1×10^{12} vg per mouse) via the intraperitoneal route. *Ex vivo* muscle force measurements were performed on EDL muscles at 5 weeks of age. (B) Specific tetanic force was significantly increased in the *mdx*/ΔIMTR and *mdx*/ΔIMTR$^+$ treated mice compared with PBS-treated *mdx* controls. Data are presented as mean ± SEM (n = 4 mice per group). Statistical significance was assessed using a two-tailed Student's t test (*$p < 0.05$). (C) Force drop after eccentric contraction (ECC) data from EDL muscles demonstrated reduced ECC force drop after a series of ECCs, from *mdx*/ΔIMTR (n = 2) and *mdx*/ΔIMTR$^+$ (n = 3) mice compared to controls (n = 3). Data are presented as mean ± SEM. Statistical significance was determined by two-way ANOVA using a repeated measures design followed by Bonferroni's multiple-comparisons test (*$p < 0.05$, **$p < 0.01$, ***$p < 0.001$).

the myotropic MyoAAV2A (as compared to using conventional AAV capsids) for utrophin editing in muscle was successful, as increased delivery to cardiac and skeletal muscle in combination with liver de-targeting was noted (Figure S4). We believe that further improvements to efficiency should be achievable by using muscle-specific promoters in combination with newer engineered myotropic capsids or the utilization of non-viral platforms for delivery. The recent demonstrations of lipid nanoparticles (LNPs) to deliver mRNA[66–68] and gene editing[69] cargoes highlight their versatility and offer a path forward for therapeutic development.[70] LNP-based delivery offers significant advantages as it can enable transient nuclease exposure, has a larger carrying capacity, and permits repeat dosing.[71] In preliminary studies, we delivered IMTR-targeting sgRNA pairs together with SpCas9 mRNA in LNP formulations to C2C12 cells and detected the expected editing product, albeit at low efficiency (Figure S7). Current experiments are underway to improve muscle utrophin editing efficiency in our laboratories. While efficient skeletal muscle targeting by LNPs remains challenging, ongoing advances in tissue-specific targeting of LNPs[72–74] provide a cause for optimism. FDA's approval of Cas9-nuclease based CASGEVY (for sickle cell disease), and the recent description of LNP-based Cas9-deaminase gene editing to treat an inherited urea cycle disorder in an infant[75] exemplify the utility of this platform.

Natural history studies of DMD patients,[3,8] along with inducible utrophin transgenic mouse data,[76] support the notion that utrophin-based strategies, such as the utrophin gene-editing approach described here, would be more effective if initiated early in the disease i.e., prior to replacement of muscle with fibro-fatty tissue. The recent addition of DMD to the recommended uniform screening panel (RUSP) in the US should allow therapies to be initiated early, prior to the occurrence of significant, irreversible muscle loss. Given that diagnosis at birth (rather than between 4 and 5 years, as is the case currently)[77] will likely become the norm for DMD diagnosis in the future, we suggest that initiation of treatment of *mdx* mice in the neonatal period as described in this study, rather than later in life, may represent a more clinically relevant time point for preclinical studies. Finally, we would note that the utrophin gene editing strategy described here could, in principle, be used in combination with several other utrophin-based or utrophin-independent strategies to potentially synergize the outcome.

MATERIALS AND METHODS

sgRNA design and cloning

All sgRNAs (sg1–sg6; Table S1) were designed using the IDT Custom Alt-R CRISPR-Cas9 guide RNA design tool. To enable AAV packaging, dual-sgRNA expression constructs were generated using a two-step molecular cloning strategy. In the first step, complementary guide DNA oligonucleotides (sg1, sg4, sg5, and sg6; Table S5) were annealed and subcloned into the BspQI site of the pAAV-U6-gRNA-CBh-mCherry plasmid (#91947, Addgene) to generate single-sgRNA expression constructs. Next, the dual-sgRNA construct pAAV-(U6-sg6-RNA)–(U6-sg4-RNA)–CBh-mCherry was generated by PCR amplification of the U6-sg4-RNA cassette from pAAV-(U6-sg4-RNA)-CBh-mCherry using the following primers: forward, 5′-AATTTCTAGAGTGAGGGCCTATTTCCCATGATT-3′; reverse, 5′-AATTGGTACCTCTAGAATTTGTCTGCAGA-3′, and the amplified fragment was subsequently subcloned into the XbaI and KpnI restriction sites of pAAV-(U6-sg6-RNA)-CBh-

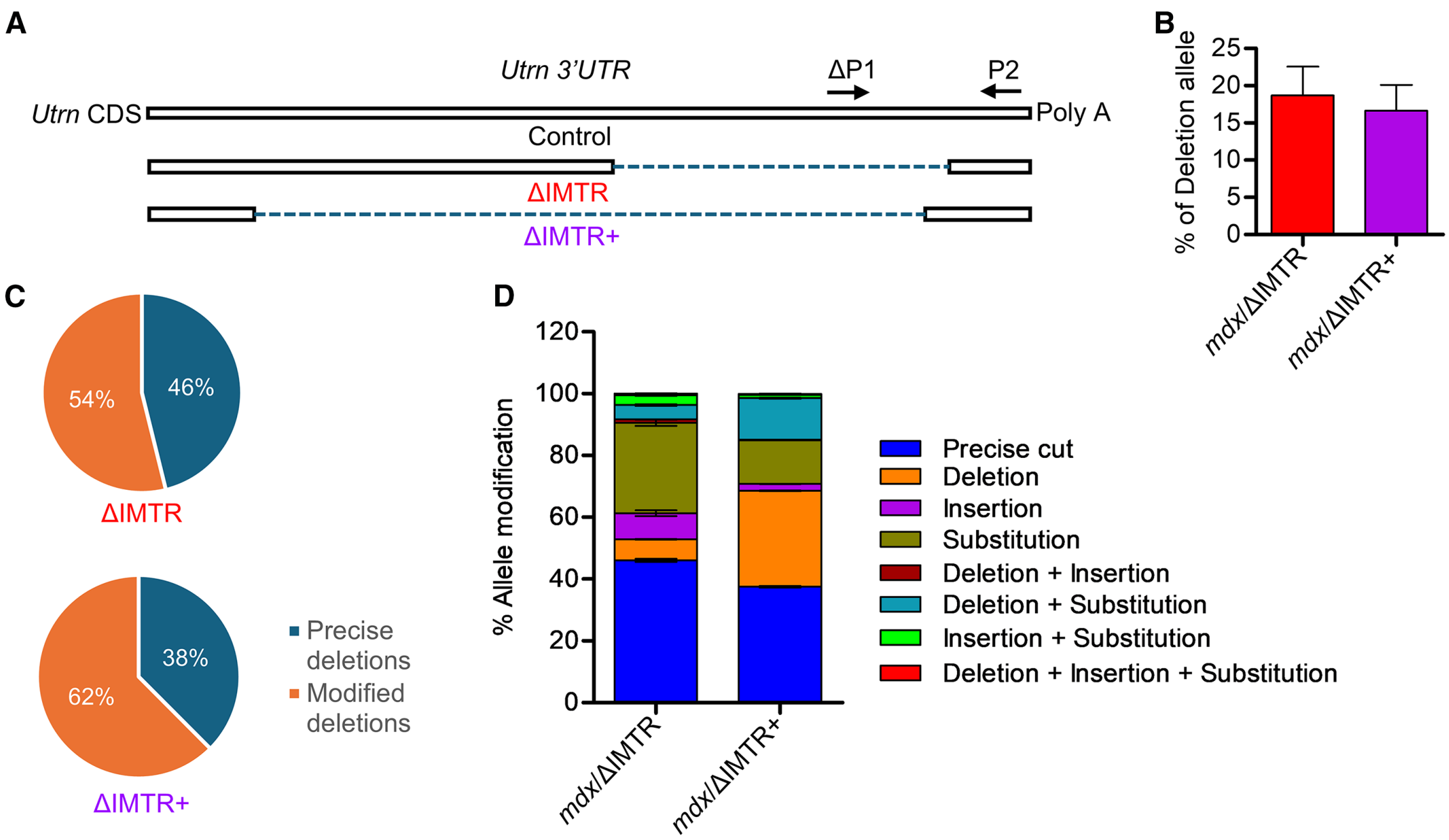

Figure 5. Characterization of *in vivo* IMTR/IMTR+ gene editing

(A) Schematic diagram illustrating the primer positions and QC-PCR strategy for quantifying editing efficiency. Primer positions within the *Utrn* 3′ UTR are shown, with the forward primer (ΔP1) located inside the deleted IMTR/IMTR+ region and the reverse primer (P2) is located downstream of the deletion site. (B) Deletion efficiency in *mdx*/ΔIMTR and *mdx*/ΔIMTR+ mouse muscle was determined by qPCR using genomic DNA isolated from gastrocnemius muscle of control and edited mice. Data are presented as mean ± SEM (*n* = 4 mice per group). (C) Pie charts showing the proportions of precise and modified deletion alleles in *mdx*/ΔIMTR and *mdx*/ΔIMTR+ mice determined by targeted amplicon deep sequencing. *n* = 4. (D) CRISPResso2 analysis of amplicon deep sequencing data. Stacked bar graphs show the frequencies of precise deletions, deletions, insertions, substitutions, deletion plus insertion, deletion plus substitution, insertion plus substitution, and combined deletion, insertion, and substitution events in *mdx*/ΔIMTR and *mdx*/ΔIMTR+ mice. Data are presented as mean ± SEM (*n* = 4 mice per group) and details are provided in Figure S3.

mCherry. Similarly, the dual-sgRNA construct pAAV-(U6-sg1-RNA)–(U6-sg5-RNA)–CBh-mCherry was generated using the same strategy. All dual-sgRNA AAV expression constructs were verified by Sanger sequencing (Genewiz, USA) prior to downstream applications.

Cell culture, transfection, and clonal selection

Mouse myoblast C2C12 cells (ATCC) were seeded in 24-well plates at a density of 3 × 10^4 cells per well. C2C12 cells were maintained in Dulbecco's modified Eagle's medium (DMEM, #10569010, Gibco) supplemented with 10% fetal bovine serum (FBS, #A5256701, Gibco) and 1% penicillin-streptomycin (#15140122, Gibco) at 37°C in a humidified incubator with 5% CO_2. Differentiated H2K *mdx* myoblasts (a gift from Professor Jennifer Morgan, University College London, UK) were used to assess genome-editing efficiency and utrophin expression following MyoAAV2A-SpCas9/sgRNA transduction. Cells were cultured on tissue culture flasks coated with 0.1 mg mL^{-1} Matrigel in growth medium consisting of DMEM (#11965092, Gibco) supplemented with 20% heat-inactivated FBS (#A5256701, Gibco), 2% L-glutamine (#25030081, Gibco), 2% chicken embryo extract (#2850145, MP Biomedicals), 1% penicillin-streptomycin, and 20 U ml^{-1} recombinant mouse interferon-γ (#315-05-100UG, Gibco). Cells were maintained at 33°C in a humidified incubator with 5% CO_2. Differentiation was induced by replacing the growth medium with differentiation medium consisting of DMEM supplemented with 2% horse serum, 2 mM L-glutamine, and 1% penicillin-streptomycin and increasing temperature to 37°C.[49] For ribonucleoprotein (RNP) delivery, dual sgRNAs were co-transfected with recombinant SpCas9 protein (Alt-R S. pyogenes Cas9 nuclease V3, #1081058, IDT) using Lipofectamine CRISPRMAX Cas9 Transfection Reagent (#CMAX00003, Invitrogen), according to the manufacturer's instructions. Edited C2C12 *Utrn* 3′ UTR clonal cell lines were generated by transfecting sgRNA pairs together with SpCas9 protein, followed by limiting dilution into 96-well plates 48 h post-transfection. Individual colonies were expanded and screened by genomic DNA isolation and PCR-based genotyping.

Validation of sgRNAs

Gene editing efficiency was validated by transfecting C2C12 cells with sgRNA pairs and recombinant SpCas9 protein. Genomic DNA was isolated 48 h post-transfection using the NucleoSpin Tissue Mini Kit (#740952.250, MACHEREY-NAGEL), and the *Utrn* 3′ UTR was amplified using primer pairs P1 (5′-GTATTGCCCTCTT CTGCAAATGC-3′) and P2 (5′-TAGATGACAGTCTGCGAT TAACCAC-3′). Successful CRISPR-Cas9-mediated deletion resulted in shorter PCR amplicons (∼330 bp, ∼450 bp, ∼850 bp and ∼1.3 kb)

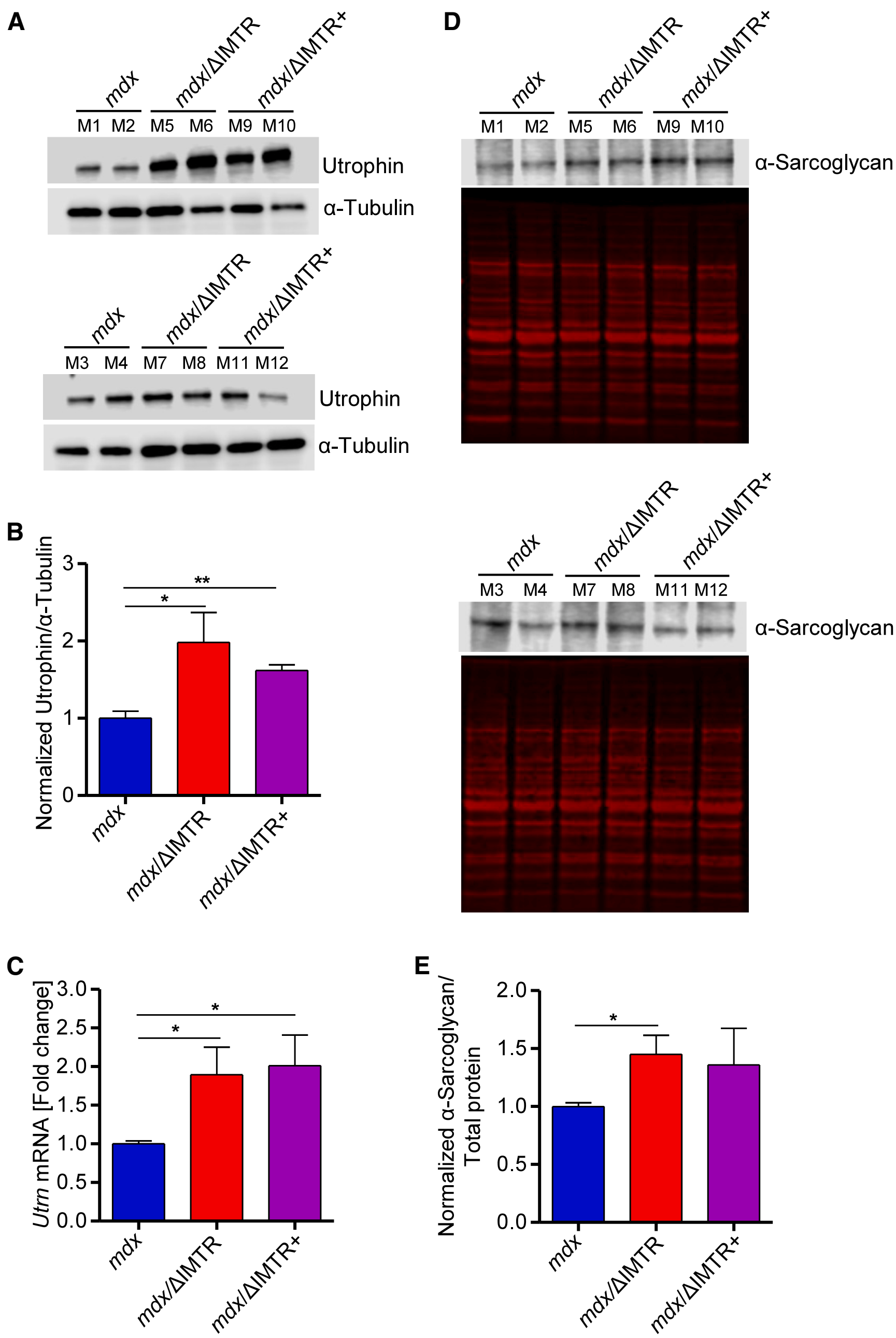

Figure 6. *In vivo* utrophin gene editing increases utrophin and α-sarcoglycan expression in *mdx* muscle

(A) Representative western blot showing utrophin expression in gastrocnemius muscle lysates from *mdx*, *mdx*/ΔIMTR, and *mdx*/ΔIMTR$^+$ mice; α-tubulin was used as a loading control. (B) Quantification of increased utrophin protein expression level in MyoAAV treated mice compared with controls, normalized to α-tubulin. Data are presented as mean ± SEM (n = 4 mice per group). Statistical significance was assessed using a two-tailed Student's t test (*p < 0.05, **p < 0.01). (C) TaqMan RT-qPCR analysis of utrophin mRNA levels in gastrocnemius muscle. Both *mdx*/ΔIMTR and *mdx*/ΔIMTR$^+$ groups showed increased expression compared with PBS-treated *mdx* controls (n = 4 mice per group). Expression was normalized to *Gapdh*. Data are presented as mean ± SEM (n = 4 mice per group). Statistical significance was assessed using a two-tailed Student's t test (*p < 0.05). (D) Representative western blot showing α-sarcoglycan expression in gastrocnemius muscle lysates from MyoAAV treated mice compared to *mdx* controls, normalized to total protein. (E) Quantification of increased α-sarcoglycan expression in MyoAAV treated mice, normalized to total protein. Data are presented as mean ± SEM (n = 4 mice per group). Statistical significance was assessed using a two-tailed Student's t test (*p < 0.05).

compared with the unedited wild-type product (∼1.9 kb), which were resolved by agarose gel electrophoresis. Deletion-derived PCR products were excised, purified using a gel extraction kit (#28704, Qiagen), and validated by Sanger sequencing (Genewiz, USA).

MyoAAV2A production

AAV transfer plasmids pAAV-(U6-sg6-RNA)–(U6-sg4-RNA)–CBh-mCherry, pAAV-(U6-sg1-RNA)–(U6-sg5-RNA)–CBh-mCherry, and pAAV-EFS-SpCas9 (#104588, Addgene) were purified using the Invitrogen PureLink HiPure Plasmid Midiprep Kit (#K210004, Invitrogen). MyoAAV2A vectors were generated by triple-plasmid transfection using the appropriate AAV transfer plasmid, MyoAAV2A capsid plasmid, and adenoviral helper plasmids to support AAV replication and packaging. Viral vectors were produced, purified, and titrated by Vector Biolabs (Malvern, PA, USA).

MyoAAV2A transduction and systemic delivery to *mdx* mice

Differentiated H2K cells were transduced with either MyoAAV2A-sg6/sg4 or MyoAAV2A-sg1/sg5 in combination with MyoAAV2A-SpCas9 at a 1:1 vector genome ratio, corresponding to a total dose of 5 × 10^8 vector genomes (vg) per well in a 24-well plate. Cells were harvested 48 h post-transduction for genomic DNA isolation using the NucleoSpin Tissue Mini Kit (#740952.250, MACHEREY-NAGEL), and protein lysates prepared as described.

Male *mdx* mice (C57BL/10ScSn-*Dmd*^{mdx}/J, strain #001801, The Jackson Laboratory) were used for systemic delivery studies. Neonatal (1 week old) mice received intraperitoneal injections of PBS or MyoAAV2A-sg6/sg4 or MyoAAV2A-sg1/sg5 in combination with MyoAAV2A-SpCas9 at a 1:1 vector genome ratio, with a total dose of 1 × 10^{12} vg per mouse (∼1.5 × 10^{14} vg/kg body weight).

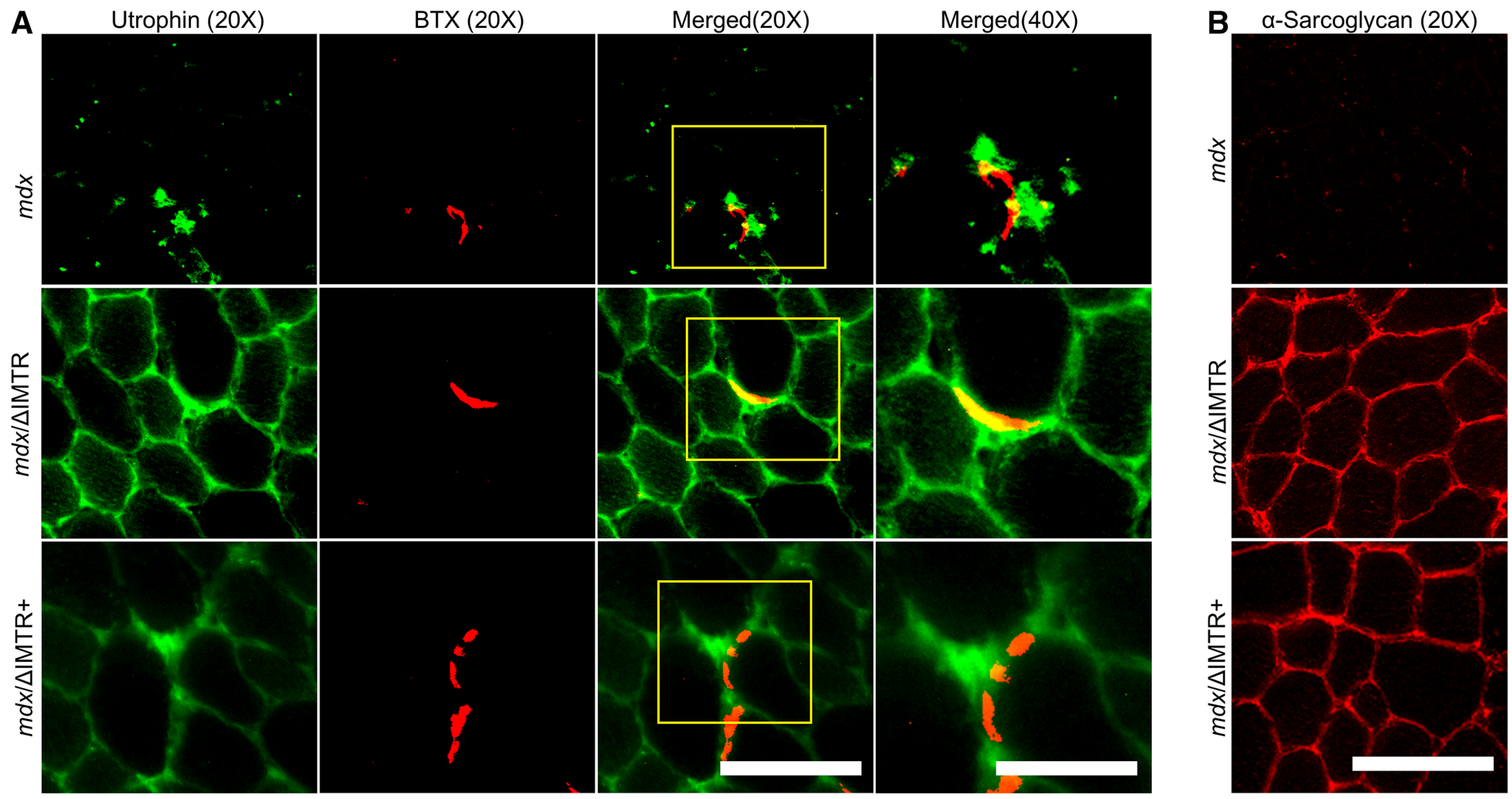

Figure 7. Systemic MyoAAV2A-Cas9/sgRNA-mediated utrophin editing increases sarcolemmal utrophin and α-sarcoglycan expression

(A) Immunofluorescence of TA muscle sections for utrophin (green) and α-bungarotoxin (red). Increased sarcolemmal utrophin localization is observed in *mdx*/ΔIMTR and *mdx*/ΔIMTR$^+$ muscles compared with PBS-treated *mdx* controls. α-Bungarotoxin marks neuromuscular junctions. Insets mark synaptic areas and are shown at higher magnifications. Scale bars, 50 μm. (20×) and 25 μm. (40×). (B) Immunofluorescence for α-sarcoglycan (red) showing increased sarcolemmal expression in *mdx*/ΔIMTR and *mdx*/ΔIMTR$^+$ muscles compared with *mdx* controls. Scale bars, 50 μm.

Vector genome ratio quantification was undertaken using standard curve genomic qPCR. Primer sequences are provided in Table S6. Mice were euthanized 4 weeks post-injection, and tissues were harvested for physiological, biochemical, and histological analyses. All animal experiments were conducted in accordance with institutional guidelines and were approved by the Institutional Animal Care and Use Committee (IACUC) of the University of Pennsylvania.

Deletion efficiency quantification

Deletion efficiency was quantified by SYBR Green-based QC-PCR method[50] using genomic DNA isolated from the heart, liver, kidney, gastrocnemius, and diaphragm of treated *mdx* mice. Primers were designed to amplify 265 bp of the unedited *Utrn* allele, with the forward primer (ΔP1) located inside the deleted IMTR/IMTR$^+$ region (1,681–1,702 nt) and the reverse primer (P2) is located downstream of the deletion site (1,945-1,921 nt) in the 3′ UTR (Primer Sequences Table S7). Amplification was normalized to genomic *Gapdh*, and deletion efficiency was calculated from the reduction in the unedited allele in edited mice relative to untreated controls using the $2^{-\Delta\Delta C_T}$ method.

Characterization of MyoAAV2A-SpCas9/sgRNAs-mediated *Utrn* gene editing *in vivo* by amplicon deep sequencing

To characterize mutations resulting from the *in vivo* gene editing, genomic DNA was isolated from gastrocnemius muscle of edited mice and amplified using Phusion High-Fidelity DNA Polymerase (#M0530S, New England Biolabs) and gene-specific primers (Table S8). Primers were designed to generate a 500-bp amplicon with the expected precise deletion junction positioned near the center of the amplicon to ensure uniform sequencing coverage across the editing site. PCR products were gel purified and subjected to paired-end next-generation sequencing (NGS) on an Illumina MiSeq platform (Azenta Life Sciences). Raw FASTQ files were analyzed using CRISPResso2[78] to quantify and classify repair outcomes, including precise deletions, insertions, deletions, substitutions, and complex editing events at the deletion junction.

Mouse muscle physiology analysis

Ex vivo functional analyses of EDL muscle force generation and force drop after ECC were performed as previously described[33,79] (https://www.treat-nmd.eu/). Following functional assessment, muscles were embedded in OCT and flash-frozen in isopentane pre-cooled in liquid nitrogen and stored at −80°C until further analyses.

Protein extraction and western blotting

Cells and tissues were lysed in ice-cold RIPA buffer (#9806, Cell Signaling Technology) supplemented with cOmplete Mini protease inhibitor cocktail (#11836153001, Roche) and 1 mM phenylmethylsulfonyl fluoride (PMSF, #52332-5GM, Sigma-Aldrich). For tissue samples, 5 mm stainless steel beads (#69989, Qiagen) were added to 1× RIPA buffer, and homogenization was performed using a TissueLyser LT (#85600, Qiagen) at 30 cycles s^{-1} for 2 min. Lysates were centrifuged at 14,000 ×*g* for 15 min at 4°C and supernatants collected. Protein concentrations were determined using the Pierce

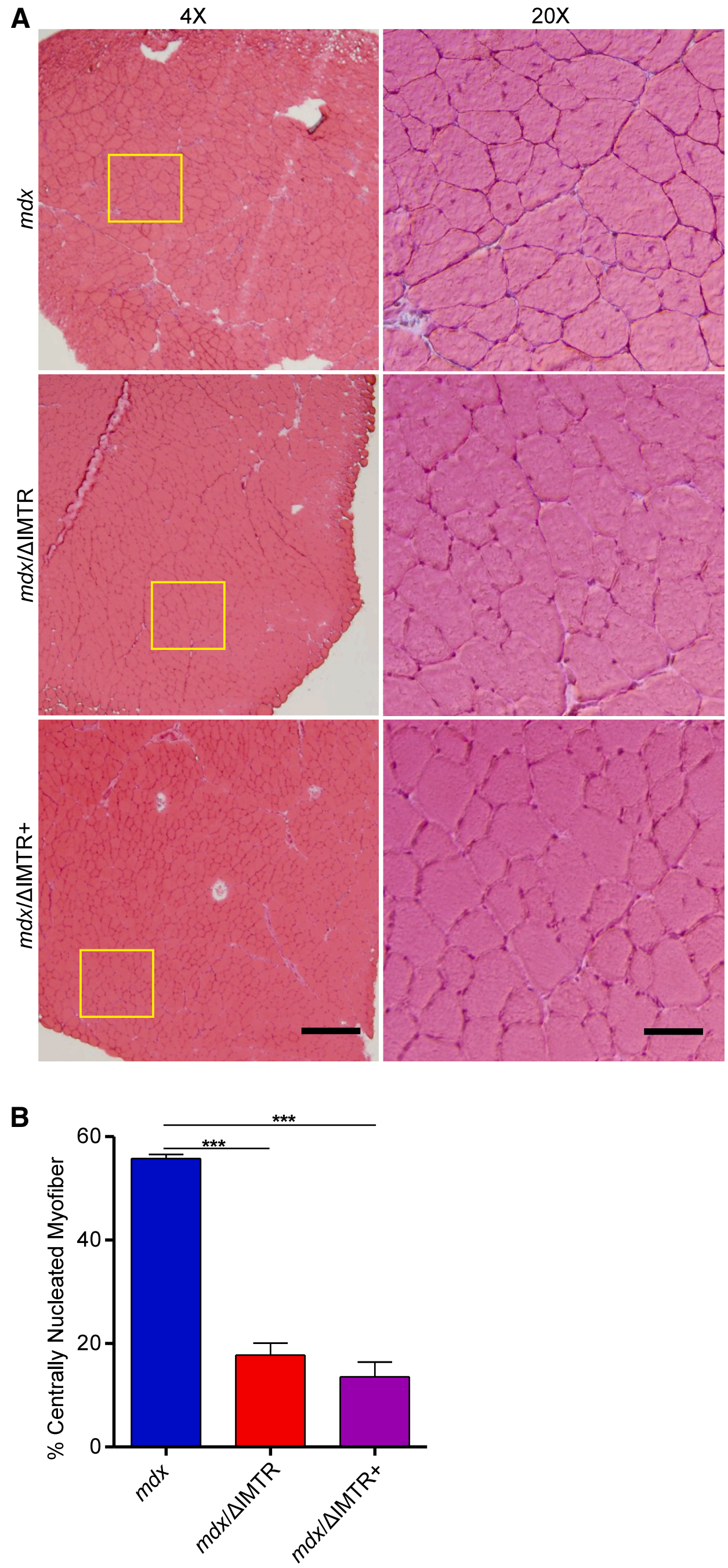

Figure 8. MyoAAV2A-Cas9/sgRNA-mediated utrophin editing improves dystrophic muscle pathology

(A) Representative hematoxylin and eosin (H&E)-stained sections of TA muscle from PBS-treated *mdx*, *mdx*/ΔIMTR, and *mdx*/ΔIMTR^{+} mice. Left images show low-magnification views (scale bars, 250 μm); inset regions are shown at higher magnification on the right (scale bars, 50 μm). (B) Quantification of the improvement of dystrophic pathology. Bar graphs show quantitation of the proportion of CNFs demonstrating a significant reduction in the proportion of CNFs in *mdx*/ΔIMTR and *mdx*/ΔIMTR^{+} muscles compared with controls. Data are presented as mean ± SEM (n = 4 mice per group). A total of 3,254 fibers were counted. Statistical significance was assessed using a two-tailed Student's t test (***$p < 0.001$).

BCA Protein Assay Kit (#23227, Thermo Fisher Scientific) according to the manufacturer's instructions. Equal amounts of protein (10 μg) were resolved on 3%–8% tris-acetate gels (NuPAGE, #EA0375BOX, Invitrogen) and transferred to nitrocellulose membranes using a Trans-Blot Turbo system (Bio-Rad). Membranes were blocked with 5% non-fat dry milk (#1706404XTU, Bio-Rad) in TBST (TBS containing 0.1% Tween 20) for 1 h at room temperature and incubated with primary antibodies for 1 h at room temperature: mouse anti-utrophin (1:100, #MANCHO3 [8A4], developed by Prof. Glenn E. Morris; Developmental Studies Hybridoma Bank, IA, USA) and mouse anti-α-tubulin (1:5,000, #T6199, Sigma-Aldrich). After washing, membranes were incubated with HRP-conjugated mouse IgGκ binding protein (1:2,500, #sc-516102, Santa Cruz Biotechnology) for 1 h at room temperature. For α-sarcoglycan detection, membranes were incubated with rabbit anti-α-sarcoglycan antibody (1:500, #PA5-106600, Invitrogen), followed by HRP-conjugated anti-rabbit secondary antibody (1:10,000, #7074S, Cell Signaling Technology). Signals were detected using Immobilon Western Chemiluminescent HRP Substrate (#WBKLS0500, Millipore) and imaged with a C-Digit Blot Scanner (LI-COR Biosciences). Band intensities were quantified using Image Studio software v.3.1 (LI-COR Biosciences). Utrophin protein levels were normalized to α-tubulin, and α-sarcoglycan levels were normalized to total protein using Revert 700 Total Protein Stain (#926–11011, LI-COR Biosciences).

RNA extraction and qPCR

Total RNA was extracted from mouse tissues using TRIzol reagent (#15596026, Invitrogen) and subjected to on-column DNase I treatment (#M0303S, NEB) using the RNeasy Mini Kit (#74104, Qiagen). Reverse transcription was performed using 1.5 μg of total RNA with the Verso cDNA Synthesis Kit (#AB1453B, Applied Biosystems), according to the manufacturer's instructions.

Utrn mRNA levels were quantified using a TaqMan Gene Expression Assay (#Mm01168866_m1, Applied Biosystems), with *Gapdh* (#Mm99999915_g1, Applied Biosystems) used as the endogenous control. Relative gene expression was calculated using the ΔΔCt method. α-Sarcoglycan mRNA expression was measured by SYBR Green-based qPCR using gene-specific primers (forward: 5′-GTTTCACCACTGTTCCATCCATGG-3′; reverse: 5′-TCTACT CGGGGAGGTAACCG-3′), with *Gapdh* serving as the internal control (forward: 5′-ATCACCATCTTCCAGGAGC-3′; reverse: 5′-CCTTCTCCATGGTGGTGAAG-3′).

Immunofluorescence

Frozen muscle sections (15 μm) from *mdx* mice were fixed in 4% paraformaldehyde (PFA; Invitrogen) for 15 min at room temperature, blocked with 10% horse serum in PBS for 1 h, and incubated overnight at 4°C with primary antibodies against utrophin (1:100; #MANCHO3 [8A4]; Developmental Studies Hybridoma Bank) and α-sarcoglycan (1:250, # PA5-106600, Invitrogen). Next, sections were washed with PBS and incubated for 1 h at room temperature with Alexa Fluor 488-conjugated goat anti-mouse or Cy3-conjugated goat anti-rabbit secondary antibodies (1:300, Jackson ImmunoResearch Laboratories). Sections were subsequently incubated with tetramethyl rhodamine-conjugated α-bungarotoxin (1:500, #T1175, Thermo Fisher Scientific) for 30 min to label neuromuscular junctions. All antibodies and α-bungarotoxin were diluted in PBS containing 3% horse serum. Images were acquired using a Leica DM6000 fluorescence microscope and analyzed using Leica software (LAS X Life Science). Comparative images were acquired and processed using identical settings.

Histology

Frozen muscle sections (15 μm) were stained with hematoxylin and eosin following standard protocols. Images were acquired using a light microscope (Olympus BX51), and CNFs were quantified as a percentage of total myofibers. Four non-overlapping fields per TA muscle section were analyzed for each group.

Statistical analysis

Data are presented as mean ± SEM, with sample sizes indicated in the figure legends. Statistical analyses were performed using Student's *t* test for pairwise comparisons or two-way ANOVA using repeated measures design in GraphPad Prism 5. A p value <0.05 was considered statistically significant.

DATA AND CODE AVAILABILITY

The authors declare that data supporting the findings of this study are available within the paper and its supplemental information files. Additionally, primary data files are being submitted to the journal and will be released upon publication.

ACKNOWLEDGMENTS

We thank Prof. Jennifer Morgan for the kind gift of the H2K (*mdx*) muscle cell line, Dr. Gordon Ruthel for guidance and help with imaging, Jessalyn Grant-Bier and Prof. Kiran Musunuru for guidance and help in undertaking the amplicon deep sequencing, and Dr. Alexandra Magold for helpful suggestions. This work was supported in part by grants from the Muscular Dystrophy Association (USA), The Dean's Innovation Fund, The Institute for Translational Medicine and Therapeutics' (ITMAT) Transdisciplinary Program in Translational Medicine and Therapeutics, The Institute of Regenerative Medicine, and The Institute for RNA Innovation of the Perelman School of Medicine at the University of Pennsylvania.

AUTHOR CONTRIBUTIONS

T.G., S.K.T.A., J.X., X.H., I.T., and L.S.C. planned and performed experiments and helped draft the manuscript. M.J.M., D.W., and T.S.K. helped with the overall design of the project and assisted with the manuscript.

DECLARATION OF INTERESTS

T.S.K. is a co-inventor and inventor on patents related to utrophin upregulation via inhibition of miRNAs targeting the IMTR by site blocking oligonucleotides and by gene editing for Duchenne muscular dystrophy (DMD). The IP is owned and managed by the Penn Center of Innovation, University of Pennsylvania.

SUPPLEMENTAL INFORMATION

Supplemental information can be found online at https://doi.org/10.1016/j.omta.2026.201857.

REFERENCES

1. Koenig, M., Hoffman, E.P., Bertelson, C.J., Monaco, A.P., Feener, C., and Kunkel, L.M. (1987). Complete cloning of the duchenne muscular dystrophy (DMD) cDNA and preliminary genomic organization of the DMD gene in normal and affected individuals. Cell *50*, 509–517. https://doi.org/10.1016/0092-8674(87)90504-6.

2. Hoffman, E.P., Brown, R.H., and Kunkel, L.M. (1987). Dystrophin: The protein product of the duchenne muscular dystrophy locus. Cell *51*, 919–928. https://doi.org/10.1016/0092-8674(87)90579-4.

3. Ryder, S., Leadley, R.M., Armstrong, N., Westwood, M., De Kock, S., Butt, T., Jain, M., and Kleijnen, J. (2017). The burden, epidemiology, costs and treatment for Duchenne muscular dystrophy: an evidence review. Orphanet J. Rare Dis. *12*, 79. https://doi.org/10.1186/s13023-017-0631-3.

4. Koenig, M., Monaco, A.P., and Kunkel, L.M. (1988). The complete sequence of dystrophin predicts a rod-shaped cytoskeletal protein. Cell *53*, 219–228. https://doi.org/10.1016/0092-8674(88)90383-2.

5. Matsumura, K., and Campbell, K.P. (1994). Dystrophin–glycoprotein complex: Its role in the molecular pathogenesis of muscular dystrophies. Muscle Nerve *17*, 2–15. https://doi.org/10.1002/mus.880170103.

6. Campbell, K.P. (1995). Three muscular dystrophies: Loss of cytoskeleton-extracellular matrix linkage. Cell *80*, 675–679. https://doi.org/10.1016/0092-8674(95)90344-5.

7. Brown, R.H., and Hoffman, E.P. (1988). Molecular biology of Duchenne muscular dystrophy. Trends Neurosci. *11*, 480–484. https://doi.org/10.1016/0166-2236(88)90006-9.

8. A. Engel, and C. Franzini-Armstrong, eds. (2004). Myology: Basic and Clinical, Third edition (McGraw-Hill, Medical Pub. Division).

9. Bushby, K., Finkel, R., Birnkrant, D.J., Case, L.E., Clemens, P.R., Cripe, L., Kaul, A., Kinnett, K., McDonald, C., Pandya, S., et al. (2010). Diagnosis and management of Duchenne muscular dystrophy, part 2: implementation of multidisciplinary care. Lancet Neurol. *9*, 177–189. https://doi.org/10.1016/S1474-4422(09)70272-8.

10. Waldrop, M.A., and Flanigan, K.M. (2019). Update in Duchenne and Becker muscular dystrophy. Curr. Opin. Neurol. *32*, 722–727. https://doi.org/10.1097/WCO.0000000000000739.

11. Bengtsson, N.E., Tasfaout, H., and Chamberlain, J.S. (2025). The road toward AAV-mediated gene therapy of Duchenne muscular dystrophy. Mol. Ther. *33*, 2035–2051. https://doi.org/10.1016/j.ymthe.2025.03.065.

12. Wilton, S.D., Fall, A.M., Harding, P.L., McClorey, G., Coleman, C., and Fletcher, S. (2007). Antisense Oligonucleotide-induced Exon Skipping Across the Human Dystrophin Gene Transcript. Mol. Ther. *15*, 1288–1296. https://doi.org/10.1038/sj.mt.6300095.

13. Cirak, S., Feng, L., Anthony, K., Arechavala-Gomeza, V., Torelli, S., Sewry, C., Morgan, J.E., and Muntoni, F. (2012). Restoration of the Dystrophin-associated Glycoprotein Complex After Exon Skipping Therapy in Duchenne Muscular Dystrophy. Mol. Ther. *20*, 462–467. https://doi.org/10.1038/mt.2011.248.

14. Mendell, J.R., Goemans, N., Lowes, L.P., Alfano, L.N., Berry, K., Shao, J., Kaye, E.M., and Mercuri, E.; Eteplirsen Study Group and Telethon Foundation DMD Italian Network (2016). Longitudinal effect of eteplirsen versus historical control on ambulation in D uchenne muscular dystrophy. Ann. Neurol. *79*, 257–271. https://doi.org/10.1002/ana.24555.

15. Bushby, K., Finkel, R., Wong, B., Barohn, R., Campbell, C., Comi, G.P., Connolly, A.M., Day, J.W., Flanigan, K.M., Goemans, N., et al. (2014). Ataluren treatment of patients with nonsense mutation dystrophinopathy. Muscle Nerve *50*, 477–487. https://doi.org/10.1002/mus.24332.

16. Duan, D. (2018). Systemic AAV Micro-dystrophin Gene Therapy for Duchenne Muscular Dystrophy. Mol. Ther. *26*, 2337–2356. https://doi.org/10.1016/j.ymthe.2018.07.011.

17. Long, C., Amoasii, L., Mireault, A.A., McAnally, J.R., Li, H., Sanchez-Ortiz, E., Bhattacharyya, S., Shelton, J.M., Bassel-Duby, R., and Olson, E.N. (2016). Postnatal genome editing partially restores dystrophin expression in a mouse model of muscular dystrophy. Science *351*, 400–403. https://doi.org/10.1126/science.aad5725.

18. Nelson, C.E., Hakim, C.H., Ousterout, D.G., Thakore, P.I., Moreb, E.A., Rivera, R.M.C., Madhavan, S., Pan, X., Ran, F.A., Yan, W.X., et al. (2016). In vivo genome editing improves muscle function in a mouse model of Duchenne muscular dystrophy. Science *351*, 403–407. https://doi.org/10.1126/science.aad5143.

19. Tabebordbar, M., Zhu, K., Cheng, J.K.W., Chew, W.L., Widrick, J.J., Yan, W.X., Maesner, C., Wu, E.Y., Xiao, R., Ran, F.A., et al. (2016). In vivo gene editing in dystrophic mouse muscle and muscle stem cells. Science *351*, 407–411. https://doi.org/10.1126/science.aad5177.

20. Young, C.S., Hicks, M.R., Ermolova, N.V., Nakano, H., Jan, M., Younesi, S., Karumbayaram, S., Kumagai-Cresse, C., Wang, D., Zack, J.A., et al. (2016). A Single CRISPR-Cas9 Deletion Strategy that Targets the Majority of DMD Patients Restores Dystrophin Function in hiPSC-Derived Muscle Cells. Cell Stem Cell *18*, 533–540. https://doi.org/10.1016/j.stem.2016.01.021.

21. Pini, V., Mariot, V., Dumonceaux, J., Counsell, J., O'Neill, H.C., Farmer, S., Conti, F., and Muntoni, F. (2022). Transiently expressed CRISPR/Cas9 induces wild-type dystrophin in vitro in DMD patient myoblasts carrying duplications. Sci. Rep. *12*, 3756. https://doi.org/10.1038/s41598-022-07671-w.

22. Mendell, J.R., Campbell, K., Rodino-Klapac, L., Sahenk, Z., Shilling, C., Lewis, S., Bowles, D., Gray, S., Li, C., Galloway, G., et al. (2010). Dystrophin Immunity in Duchenne's Muscular Dystrophy. N. Engl. J. Med. *363*, 1429–1437. https://doi.org/10.1056/NEJMoa1000228.

23. Aartsma-Rus, A., Van Deutekom, J.C.T., Fokkema, I.F., Van Ommen, G.B., and Den Dunnen, J.T. (2006). Entries in the Leiden Duchenne muscular dystrophy mutation database: An overview of mutation types and paradoxical cases that confirm the reading-frame rule. Muscle Nerve *34*, 135–144. https://doi.org/10.1002/mus.20586.

24. Love, D.R., Hill, D.F., Dickson, G., Spurr, N.K., Byth, B.C., Marsden, R.F., Walsh, F.S., Edwards, Y.H., and Davies, K.E. (1989). An autosomal transcript in skeletal muscle with homology to dystrophin. Nature *339*, 55–58. https://doi.org/10.1038/339055a0.

25. Khurana, T.S., Hoffman, E.P., and Kunkel, L.M. (1990). Identification of a chromosome 6-encoded dystrophin-related protein. J. Biol. Chem. *265*, 16717–16720. https://doi.org/10.1016/s0021-9258(17)44816-2.

26. Tinsley, J.M., Blake, D.J., Roche, A., Fairbrother, U., Riss, J., Byth, B.C., Knight, A.E., Kendrick-Jones, J., Suthers, G.K., Love, D.R., et al. (1992). Primary structure of dystrophin-related protein. Nature *360*, 591–593. https://doi.org/10.1038/360591a0.

27. Love, D.R., Morris, G.E., Ellis, J.M., Fairbrother, U., Marsden, R.F., Bloomfield, J.F., Edwards, Y.H., Slater, C.P., Parry, D.J., and Davies, K.E. (1991). Tissue distribution of the dystrophin-related gene product and expression in the mdx and dy mouse. Proc. Natl. Acad. Sci. USA *88*, 3243–3247. https://doi.org/10.1073/pnas.88.8.3243.

28. Tinsley, J.M., Potter, A.C., Phelps, S.R., Fisher, R., Trickett, J.I., and Davies, K.E. (1996). Amelioration of the dystrophic phenotype of mdx mice using a truncated utrophin transgene. Nature *384*, 349–353. https://doi.org/10.1038/384349a0.

29. Tinsley, J., Deconinck, N., Fisher, R., Kahn, D., Phelps, S., Gillis, J.-M., and Davies, K. (1998). Expression of full-length utrophin prevents muscular dystrophy in mdx mice. Nat. Med. *4*, 1441–1444. https://doi.org/10.1038/4033.

30. Song, Y., Morales, L., Malik, A.S., Mead, A.F., Greer, C.D., Mitchell, M.A., Petrov, M.T., Su, L.T., Choi, M.E., Rosenblum, S.T., et al. (2019). Non-immunogenic utrophin gene therapy for the treatment of muscular dystrophy animal models. Nat. Med. *25*, 1505–1511. https://doi.org/10.1038/s41591-019-0594-0.

31. Gramolini, A.O., Bélanger, G., Thompson, J.M., Chakkalakal, J.V., and Jasmin, B.J. (2001). Increased expression of utrophin in a slow vs. a fast muscle involves post-transcriptional events. Am. J. Physiol. Cell Physiol. *281*, C1300–C1309. https://doi.org/10.1152/ajpcell.2001.281.4.C1300.

32. Baby, S.M., Bogdanovich, S., Willmann, G., Basu, U., Lozynska, O., and Khurana, T.S. (2010). Differential expression of utrophin-A and -B promoters in the central nervous system (CNS) of normal and dystrophic mdx mice. Brain Pathol. *20*, 323–342. https://doi.org/10.1111/j.1750-3639.2009.00275.x.

33. Krag, T.O.B., Bogdanovich, S., Jensen, C.J., Fischer, M.D., Hansen-Schwartz, J., Javazon, E.H., Flake, A.W., Edvinsson, L., and Khurana, T.S. (2004). Heregulin ameliorates the dystrophic phenotype in *mdx* mice. Proc. Natl. Acad. Sci. USA *101*, 13856–13860. https://doi.org/10.1073/pnas.0405972101.

34. Moorwood, C., Lozynska, O., Suri, N., Napper, A.D., Diamond, S.L., and Khurana, T.S. (2011). Drug Discovery for Duchenne Muscular Dystrophy via Utrophin Promoter Activation Screening. PLoS One *6*, e26169. https://doi.org/10.1371/journal.pone.0026169.

35. Tinsley, J.M., Fairclough, R.J., Storer, R., Wilkes, F.J., Potter, A.C., Squire, S.E., Powell, D.S., Cozzoli, A., Capogrosso, R.F., Lambert, A., et al. (2011). Daily Treatment with SMTC1100, a Novel Small Molecule Utrophin Upregulator,

Dramatically Reduces the Dystrophic Symptoms in the mdx Mouse. PLoS One *6*, e19189. https://doi.org/10.1371/journal.pone.0019189.

36. Mattei, E., Corbi, N., Di Certo, M.G., Strimpakos, G., Severini, C., Onori, A., Desantis, A., Libri, V., Buontempo, S., Floridi, A., et al. (2007). Utrophin Up-Regulation by an Artificial Transcription Factor in Transgenic Mice. PLoS One *2*, e774. https://doi.org/10.1371/journal.pone.0000774.

37. Pisani, C., Strimpakos, G., Gabanella, F., Di Certo, M.G., Onori, A., Severini, C., Luvisetto, S., Farioli-Vecchioli, S., Carrozzo, I., Esposito, A., et al. (2018). Utrophin up-regulation by artificial transcription factors induces muscle rescue and impacts the neuromuscular junction in mdx mice. Biochim. Biophys. Acta Mol. Basis Dis. *1864*, 1172–1182. https://doi.org/10.1016/j.bbadis.2018.01.030.

38. Dennis, C.L., Tinsley, J.M., Deconinck, A.E., and Davies, K.E. (1996). Molecular and Functional Analysis of the Utrophin Promoter. Nucleic Acids Res. *24*, 1646–1652. https://doi.org/10.1093/nar/24.9.1646.

39. Basu, U., Lozynska, O., Moorwood, C., Patel, G., Wilton, S.D., and Khurana, T.S. (2011). Translational Regulation of Utrophin by miRNAs. PLoS One *6*, e29376. https://doi.org/10.1371/journal.pone.0029376.

40. Amirouche, A., Tadesse, H., Miura, P., Bélanger, G., Lunde, J.A., Côté, J., and Jasmin, B.J. (2014). Converging pathways involving microRNA-206 and the RNA-binding protein KSRP control post-transcriptionally utrophin A expression in skeletal muscle. Nucleic Acids Res. *42*, 3982–3997. https://doi.org/10.1093/nar/gkt1350.

41. Mishra, M.K., Loro, E., Sengupta, K., Wilton, S.D., and Khurana, T.S. (2017). Functional improvement of dystrophic muscle by repression of utrophin: let-7c interaction. PLoS One *12*, e0182676. https://doi.org/10.1371/journal.pone.0182676.

42. Ebert, M.S., Neilson, J.R., and Sharp, P.A. (2007). MicroRNA sponges: competitive inhibitors of small RNAs in mammalian cells. Nat. Methods *4*, 721–726. https://doi.org/10.1038/nmeth1079.

43. Bulaklak, K., Xiao, B., Qiao, C., Li, J., Patel, T., Jin, Q., Li, J., and Xiao, X. (2018). MicroRNA-206 Downregulation Improves Therapeutic Gene Expression and Motor Function in mdx Mice. Mol. Ther. Nucleic Acids *12*, 283–293. https://doi.org/10.1016/j.omtn.2018.05.011.

44. Sengupta, K., Mishra, M.K., Loro, E., Spencer, M.J., Pyle, A.D., and Khurana, T.S. (2020). Genome Editing-Mediated Utrophin Upregulation in Duchenne Muscular Dystrophy Stem Cells. Mol. Ther. Nucleic Acids *22*, 500–509. https://doi.org/10.1016/j.omtn.2020.08.031.

45. Soblechero-Martín, P., Albiasu-Arteta, E., Anton-Martinez, A., De La Puente-Ovejero, L., Garcia-Jimenez, I., González-Iglesias, G., Larrañaga-Aiestaran, I., López-Martínez, A., Poyatos-García, J., Ruiz-Del-Yerro, E., et al. (2021). Duchenne muscular dystrophy cell culture models created by CRISPR/Cas9 gene editing and their application in drug screening. Sci. Rep. *11*, 18188. https://doi.org/10.1038/s41598-021-97730-5.

46. Ralu, M., Guiraud, S., Dastidar, S., Galbiati, P., Sadaoui, E., Mazed, F., Amor, F., De Cian, A., Richard, I., Mamchaoui, K., et al. (2026). CRISPR-Cas9-mediated upregulation of utrophin ameliorates Duchenne muscular dystrophy. Mol. Ther. *34*, 3903–3923. https://doi.org/10.1016/j.ymthe.2026.03.025.

47. Tabebordbar, M., Lagerborg, K.A., Stanton, A., King, E.M., Ye, S., Tellez, L., Krunnfusz, A., Tavakoli, S., Widrick, J.J., Messemer, K.A., et al. (2021). Directed evolution of a family of AAV capsid variants enabling potent muscle-directed gene delivery across species. Cell *184*, 4919–4938.e22. https://doi.org/10.1016/j.cell.2021.08.028.

48. Cradick, T.J., Qiu, P., Lee, C.M., Fine, E.J., and Bao, G. (2014). COSMID: A Web-based Tool for Identifying and Validating CRISPR/Cas Off-target Sites. Mol. Ther. Nucleic Acids *3*, e214. https://doi.org/10.1038/mtna.2014.64.

49. Morgan, J.E., Beauchamp, J.R., Pagel, C.N., Peckham, M., Ataliotis, P., Jat, P.S., Noble, M.D., Farmer, K., and Partridge, T.A. (1994). Myogenic Cell Lines Derived from Transgenic Mice Carrying a Thermolabile T Antigen: A Model System for the Derivation of Tissue-Specific and Mutation-Specific Cell Lines. Dev. Biol. *162*, 486–498. https://doi.org/10.1006/dbio.1994.1103.

50. Pulido-Quetglas, C., Aparicio-Prat, E., Arnan, C., Polidori, T., Hermoso, T., Palumbo, E., Ponomarenko, J., Guigo, R., and Johnson, R. (2017). Scalable Design of Paired CRISPR Guide RNAs for Genomic Deletion. PLoS Comput. Biol. *13*, e1005341. https://doi.org/10.1371/journal.pcbi.1005341.

51. Roberds, S.L., Anderson, R.D., Ibraghimov-Beskrovnaya, O., and Campbell, K.P. (1993). Primary structure and muscle-specific expression of the 50-kDa dystrophin-associated glycoprotein (adhalin). J. Biol. Chem. *268*, 23739–23742. https://doi.org/10.1016/S0021-9258(20)80440-2.

52. Long, C., McAnally, J.R., Shelton, J.M., Mireault, A.A., Bassel-Duby, R., and Olson, E.N. (2014). Prevention of muscular dystrophy in mice by CRISPR/Cas9–mediated editing of germline DNA. Science *345*, 1184–1188. https://doi.org/10.1126/science.1254445.

53. Amoasii, L., Hildyard, J.C.W., Li, H., Sanchez-Ortiz, E., Mireault, A., Caballero, D., Harron, R., Stathopoulou, T.-R., Massey, C., Shelton, J.M., et al. (2018). Gene editing restores dystrophin expression in a canine model of Duchenne muscular dystrophy. Science *362*, 86–91. https://doi.org/10.1126/science.aau1549.

54. Ousterout, D.G., Kabadi, A.M., Thakore, P.I., Majoros, W.H., Reddy, T.E., and Gersbach, C.A. (2015). Multiplex CRISPR/Cas9-based genome editing for correction of dystrophin mutations that cause Duchenne muscular dystrophy. Nat. Commun. *6*, 6244. https://doi.org/10.1038/ncomms7244.

55. Nelson, C.E., Wu, Y., Gemberling, M.P., Oliver, M.L., Waller, M.A., Bohning, J.D., Robinson-Hamm, J.N., Bulaklak, K., Castellanos Rivera, R.M., Collier, J.H., et al. (2019). Long-term evaluation of AAV-CRISPR genome editing for Duchenne muscular dystrophy. Nat. Med. *25*, 427–432. https://doi.org/10.1038/s41591-019-0344-3.

56. Chemello, F., Chai, A.C., Li, H., Rodriguez-Caycedo, C., Sanchez-Ortiz, E., Atmanli, A., Mireault, A.A., Liu, N., Bassel-Duby, R., and Olson, E.N. (2021). Precise correction of Duchenne muscular dystrophy exon deletion mutations by base and prime editing. Sci. Adv. *7*, eabg4910. https://doi.org/10.1126/sciadv.abg4910.

57. Wojtal, D., Kemaladewi, D.U., Malam, Z., Abdullah, S., Wong, T.W.Y., Hyatt, E., Baghestani, Z., Pereira, S., Stavropoulos, J., Mouly, V., et al. (2016). Spell Checking Nature: Versatility of CRISPR/Cas9 for Developing Treatments for Inherited Disorders. Am. J. Hum. Genet. *98*, 90–101. https://doi.org/10.1016/j.ajhg.2015.11.012.

58. Liao, H.-K., Hatanaka, F., Araoka, T., Reddy, P., Wu, M.-Z., Sui, Y., Yamauchi, T., Sakurai, M., O'Keefe, D.D., Núñez-Delicado, E., et al. (2017). In Vivo Target Gene Activation via CRISPR/Cas9-Mediated Trans-epigenetic Modulation. Cell *171*, 1495–1507.e15. https://doi.org/10.1016/j.cell.2017.10.025.

59. Sengupta, K., Loro, E., and Khurana, T.S. (2020). PMO-based let-7c site blocking oligonucleotide (SBO) mediated utrophin upregulation in mdx mice, a therapeutic approach for Duchenne muscular dystrophy (DMD). Sci. Rep. *10*, 21492. https://doi.org/10.1038/s41598-020-76338-1.

60. Amoasii, L., Li, H., Zhang, Y., Min, Y.-L., Sanchez-Ortiz, E., Shelton, J.M., Long, C., Mireault, A.A., Bhattacharyya, S., McAnally, J.R., et al. (2019). In vivo non-invasive monitoring of dystrophin correction in a new Duchenne muscular dystrophy reporter mouse. Nat. Commun. *10*, 4537. https://doi.org/10.1038/s41467-019-12335-x.

61. Cong, L., Ran, F.A., Cox, D., Lin, S., Barretto, R., Habib, N., Hsu, P.D., Wu, X., Jiang, W., Marraffini, L.A., and Zhang, F. (2013). Multiplex Genome Engineering Using CRISPR/Cas Systems. Science *339*, 819–823. https://doi.org/10.1126/science.1231143.

62. Mingozzi, F., and High, K.A. (2017). Overcoming the Host Immune Response to Adeno-Associated Virus Gene Delivery Vectors: The Race Between Clearance, Tolerance, Neutralization, and Escape. Annu. Rev. Virol. *4*, 511–534. https://doi.org/10.1146/annurev-virology-101416-041936.

63. Hinderer, C., Katz, N., Buza, E.L., Dyer, C., Goode, T., Bell, P., Richman, L.K., and Wilson, J.M. (2018). Severe Toxicity in Nonhuman Primates and Piglets Following High-Dose Intravenous Administration of an Adeno-Associated Virus Vector Expressing Human SMN. Hum. Gene Ther. *29*, 285–298. https://doi.org/10.1089/hum.2018.015.

64. Ertl, H.C.J. (2022). Immunogenicity and toxicity of AAV gene therapy. Front. Immunol. *13*, 975803. https://doi.org/10.3389/fimmu.2022.975803.

65. Duan, D. (2023). Lethal immunotoxicity in high-dose systemic AAV therapy. Mol. Ther. *31*, 3123–3126. https://doi.org/10.1016/j.ymthe.2023.10.015.

66. Polack, F.P., Thomas, S.J., Kitchin, N., Absalon, J., Gurtman, A., Lockhart, S., Perez, J.L., Pérez Marc, G., Moreira, E.D., Zerbini, C., et al. (2020). Safety and Efficacy of the

BNT162b2 mRNA Covid-19 Vaccine. N. Engl. J. Med. *383*, 2603–2615. https://doi.org/10.1056/NEJMoa2034577.

67. Pardi, N., Hogan, M.J., Porter, F.W., and Weissman, D. (2018). mRNA vaccines — a new era in vaccinology. Nat. Rev. Drug Discov. *17*, 261–279. https://doi.org/10.1038/nrd.2017.243.

68. Baden, L.R., El Sahly, H.M., Essink, B., Kotloff, K., Frey, S., Novak, R., Diemert, D., Spector, S.A., Rouphael, N., Creech, C.B., et al. (2021). Efficacy and Safety of the mRNA-1273 SARS-CoV-2 Vaccine. N. Engl. J. Med. *384*, 403–416. https://doi.org/10.1056/NEJMoa2035389.

69. Gillmore, J.D., Gane, E., Taubel, J., Kao, J., Fontana, M., Maitland, M.L., Seitzer, J., O'Connell, D., Walsh, K.R., Wood, K., et al. (2021). CRISPR-Cas9 In Vivo Gene Editing for Transthyretin Amyloidosis. N. Engl. J. Med. *385*, 493–502. https://doi.org/10.1056/NEJMoa2107454.

70. Parhiz, H., Atochina-Vasserman, E.N., and Weissman, D. (2024). mRNA-based therapeutics: looking beyond COVID-19 vaccines. Lancet *403*, 1192–1204. https://doi.org/10.1016/S0140-6736(23)02444-3.

71. Kenjo, E., Hozumi, H., Makita, Y., Iwabuchi, K.A., Fujimoto, N., Matsumoto, S., Kimura, M., Amano, Y., Ifuku, M., Naoe, Y., et al. (2021). Low immunogenicity of LNP allows repeated administrations of CRISPR-Cas9 mRNA into skeletal muscle in mice. Nat. Commun. *12*, 7101. https://doi.org/10.1038/s41467-021-26714-w.

72. Chen, J., Xu, Y., Zhou, M., Xu, S., Varley, A.J., Golubovic, A., Lu, R.X.Z., Wang, K.C., Yeganeh, M., Vosoughi, D., and Li, B. (2023). Combinatorial design of ionizable lipid nanoparticles for muscle-selective mRNA delivery with minimized off-target effects. Proc. Natl. Acad. Sci. USA *120*, e2309472120. https://doi.org/10.1073/pnas.2309472120.

73. Ji, J., Lipkow, E., Anton, N., Crucifix, C., Eberling, P., and Laporte, J. (2026). Myomerger-derived peptide enhances skeletal muscle tropism and reduces liver transduction of lipid nanoparticles for gene delivery. Mol. Ther. Nucleic Acids *37*, 102785. https://doi.org/10.1016/j.omtn.2025.102785.

74. Hamilton, A.G., Thatte, A.S., Xu, J., Luo, Z., Safford, H.C., Swingle, K.L., Muscat-Rivera, J., Kegel, M., Han, X., Joseph, R.A., et al. (2026). High-Throughput In Vivo Screening Using Barcoded mRNA Identifies Lipid Nanoparticles With Extrahepatic Tropism for In Situ Immunoengineering. Adv. Mater. *38*, e14370. https://doi.org/10.1002/adma.202514370.

75. Musunuru, K., Grandinette, S.A., Wang, X., Hudson, T.R., Briseno, K., Berry, A.M., Hacker, J.L., Hsu, A., Silverstein, R.A., Hille, L.T., et al. (2025). Patient-Specific In Vivo Gene Editing to Treat a Rare Genetic Disease. N. Engl. J. Med. *392*, 2235–2243. https://doi.org/10.1056/NEJMoa2504747.

76. Squire, S., Raymackers, J.M., Vandebrouck, C., Potter, A., Tinsley, J., Fisher, R., Gillis, J.M., and Davies, K.E. (2002). Prevention of pathology in mdx mice by expression of utrophin: analysis using an inducible transgenic expression system. Hum. Mol. Genet. *11*, 3333–3344. https://doi.org/10.1093/hmg/11.26.3333.

77. Mercuri, E., Pane, M., Cicala, G., Brogna, C., and Ciafaloni, E. (2023). Detecting early signs in Duchenne muscular dystrophy: comprehensive review and diagnostic implications. Front. Pediatr. *11*, 1276144. https://doi.org/10.3389/fped.2023.1276144.

78. Clement, K., Rees, H., Canver, M.C., Gehrke, J.M., Farouni, R., Hsu, J.Y., Cole, M.A., Liu, D.R., Joung, J.K., Bauer, D.E., and Pinello, L. (2019). CRISPResso2 provides accurate and rapid genome editing sequence analysis. Nat. Biotechnol. *37*, 224–226. https://doi.org/10.1038/s41587-019-0032-3.

79. Bogdanovich, S., Krag, T.O.B., Barton, E.R., Morris, L.D., Whittemore, L.-A., Ahima, R.S., and Khurana, T.S. (2002). Functional improvement of dystrophic muscle by myostatin blockade. Nature *420*, 418–421. https://doi.org/10.1038/nature01154.

OMTA, Volume *34*

Supplemental information

Functional improvement of dystrophic muscle by utrophin gene editing

Trinath Ghosh, SK Tousif Ahamed, Junchao Xu, Xuexiang Han, Istvan Tombacz, Lesley S. Chaboub, Michael J. Mitchell, Drew Weissman, and Tejvir S. Khurana

Supplemental Methods

Vector genome quantification

Vector genome copy number was quantified by SYBR Green–based quantitative PCR (qPCR) using primer pairs specific to each sgRNA cassette (Table S6). For the ΔIMTR (sg6/sg4) vector construct, primer pair F1/R was used, whereas primer pair F2/R was used for $\Delta IMTR^{+}$(sg1/sg5) vector construct detection. Genomic DNA was isolated from heart, liver, kidney, gastrocnemius, and diaphragm tissues collected from treated and control *mdx* mice and used as the qPCR template. Standard curves were generated using gel-purified PCR amplicons corresponding to each target sequence, enabling absolute quantification of vector genome copies.

In Vitro transcribed mRNA production

The plasmid encoding codon-optimized Cas9, flanked between optimized untranslated regions and containing a 101-base poly(A) tail, was supplied by GenScript (Piscataway, NJ, USA) as 1 mg/ml industrial-grade, endotoxin-free plasmid in nuclease-free water. The plasmid was linearized, purified and subjected to *in vitro* transcription using MEGAscript T7 transcription kits (Thermo Fisher Scientific, Waltham, MA, USA) and co-transcriptional capping with CleanCap (TriLink Biotechnologies, San Diego, CA, USA). Uridines were fully substituted with N1-methylpseudouridine (TriLink Biotechnologies) [1–3] mRNA was cellulose-purified to remove double stranded contaminants, as previously described.[4]

LNP formulation

LNPs were formulated by microfluidic mixing using an ionizable lipid (5D8), DOPE, cholesterol, and DMG-PEG2000 at a molar ratio of 40:10:48.5:1.5. Lipids were dissolved in ethanol and mixed with RNA diluted in 10 mM citrate buffer (pH 3.0) at an ethanol-to-aqueous flow rate ratio of 1:3. The ionizable lipid-to-RNA ratio was maintained at 10:1 (w/w). For *Utrn* gene-editing cargo formulations, Cas9 mRNA and two sgRNAs were encapsulated at a weight ratio of 2:1:1 (Cas9 mRNA:sgRNA1:sgRNA2). Following formulation, LNPs were dialyzed into 1× PBS using a 20 kDa molecular weight cut-off membrane for 2 h, sterile filtered through a 0.22 µm filter, and stored at 4 °C until use. The resulting LNPs typically exhibited a particle diameter of ~80–120 nm with RNA encapsulation efficiency exceeding 90%.

LNP and sgRNAs/SpCas9 mRNA transfection

For LNP–based delivery, LNP formulations were added directly to C2C12 cells plated in 24-well plates. For mRNA-based delivery, dual sgRNAs and SpCas9 mRNA were co-transfected using Lipofectamine™ MessengerMAX™ Transfection Reagent (#LMRNA001, Invitrogen) following the manufacturer's protocol. Cells were harvested 48 h post-transfection for genomic DNA isolation.

Supplemental Tables

Table S1. Single guide RNAs used for *in vitro* transfection.

No.	Name of sgRNA	**Sequence (5'-3')**	**gRNA/PAM Orientation**
1	sg1	mG*mC*mU* rUrArC rUrGrG rGrUrG rUrUrC rUrArC rUrGrG rUrUrU rUrArG rArGrC rUrArG rArArA rUrArG rCrArA rGrUrU rArArA rArUrA rArGrG rCrUrA rGrUrC rCrGrU rUrArU rCrArA rCrUrU rGrArA rArArA rGrUrG rGrCrA rCrCrG rArGrU rCrGrG rUrGrC mU*mU*mU* rU	GCTTACTGGGTGTTCTACTG/AGG Forward
2	sg2	mA*mG*mG* rArArU rGrUrA rArUrA rArUrG rUrArA rArGrG rUrUrU rUrArG rArGrC rUrArG rArArA rUrArG rCrArA rGrUrU rArArA rArUrA rArGrG rCrUrA rGrUrC rCrGrU rUrArU rCrArA rCrUrU rGrArA rArArA rGrUrG rGrCrA rCrCrG rArGrU rCrGrG rUrGrC mU*mU*mU* rU	AGGAATGTAATAATGTAAAG/TGG Reverse
3	sg3	mC*mA*mC* rCrArU rCrCrU rUrUrA rGrUrA rArCrC rArCrG rUrUrU rUrArG rArGrC rUrArG rArArA rUrArG rCrArA rGrUrU rArArA rArUrA rArGrG rCrUrA rGrUrC rCrGrU rUrArU rCrArA rCrUrU rGrArA rArArA rGrUrG rGrCrA rCrCrG rArGrU rCrGrG rUrGrC mU*mU*mU* rU	CACCATCCTTTAGTAACCAC/TGG Forward
4	sg4	mG*mA*mG* rUrGrU rGrUrU rCrArA rCrUrA rGrGrU rArUrG rUrUrU rUrArG rArGrC rUrArG rArArA rUrArG rCrArA rGrUrU rArArA rArUrA rArGrG rCrUrA rGrUrC rCrGrU rUrArU rCrArA rCrUrU rGrArA rArArA rGrUrG rGrCrA rCrCrG rArGrU rCrGrG rUrGrC mU*mU*mU* rU	GAGTGTGTTCAACTAGGTAT/TGG Forward
5	sg5	mA*mC*mG* rArCrA rCrCrA rArArU rCrUrA rCrArA rCrUrG rUrUrU rUrArG rArGrC rUrArG rArArA rUrArG rCrArA rGrUrU rArArA rArUrA rArGrG rCrUrA rGrUrC rCrGrU rUrArU rCrArA rCrUrU rGrArA rArArA rGrUrG rGrCrA rCrCrG rArGrU rCrGrG rUrGrC mU*mU*mU* rU	ACGACACCAAATCTACAACT/TGG Reverse
6	sg6	mC*mC*mU* rCrUrU rArUrU rUrArU rUrArC rUrCrA rGrArG rUrUrU rUrArG rArGrC rUrArG rArArA rUrArG rCrArA rGrUrU rArArA rArUrA rArGrG rCrUrA rGrUrC rCrGrU rUrArU rCrArA rCrUrU rGrArA rArArA rGrUrG rGrCrA rCrCrG rArGrU rCrGrG rUrGrC mU*mU*mU* rU	CCTCTTATTTATTACTCAGA/AGG Reverse

* 2′-O-methyl + phosphorothioate modification

Table S2. Primers used to amplify unedited wild type and deletion mutant alleles of *Utrn* 3'UTR.

Primers	Orientation	Sequence (5'-3')
P1	Forward	GTATTGCCCTCTTCTGCAAATGC
P2	Reverse	TAGATGACAGTCTGCGATTAACCAC

Table S3. *Ex-vivo* functional assessment of EDL.

	n	*mdx*	*mdx*/ΔIMTR	*mdx*/ΔIMTR$^+$
**Twitch force (mN)**	4	25.58 ± 3.973	30.83 ± 2.901, P=0.3272	26.07 ± 3.504, P=0.9293
**Specific twitch force (mN/mm^2)**	4	31.54 ± 4.286	41.17 ± 4.196, P=0.1595	36.51 ± 3.549, P=0.4059
**Tetanic force (mN)**	4	136.4 ± 10.71	165.5 ± 17.98, P=0.2141	156.9 ± 24.84, P=0.4765
**Specific tetanic force (mN/mm^2)**	4	169.1 ± 10.42	217.6 ± 12.77, P=0.0257*	214.9 ± 14.14, P=0.04*
**ECC force drop (Contraction 1-7, %)**	3, 2, 3	19.24 ± 4	7.23 ± 1.5, P<0.01**	7.68 ± 0.87, P<0.001***
**L0 (mm)**	4	10.48 ± 0.85	10.03 ± 0.31, P=0.6372	10.25 ± 0.32, P=0.8133
**Muscle Mass (mg)**	4	8.84 ± 0.26	8.10 ± 0.81, P=0.4202	7.88 ± 1.13, P=0.4360
**CSA (mm^2)**	4	0.81 ± 0.07	0.73 ± 0.12, P=0.5576	0.76 ± 0.06, P=0.5686
**CK (U/L)**	4+	23510 ± 10510	7691 ± 2896, P=0.1971	7031 ± 1924, P=0.1741

CSA, cross sectional area; ECC, eccentric contraction; L0, muscle length; +samples lysed; two-tailed Student's t-test (*P < 0.05, **P < 0.01, ***P < 0.001)

Table S4. Deletion efficiency following AAV transduction.

		Mean ± SEM	
**Tissue**	**n**	**% of ΔIMTR**	**% of ΔIMTR$^+$**
**Heart**	4,3	15.42 ± 4.35	16.14 ± 2.42
**Kidney**	4	12.80 ± 3.07	12.04 ± 3.43
**Liver**	4	10.87 ± 1.16	12.71 ± 2.49
**Gastrocnemius**	4	18.70 ± 3.84	16.63 ± 3.45
**Diaphragm**	4	20.13 ± 5.87	15.36 ± 3.87

Note: Heart n=4 for ΔIMTR and n=3 for ΔIMTR$^+$

Table S5. Complementary DNA oligonucleotides for cloning and expression of sgRNA

Oligonucleotides	Sequence (5'-3')
sg1_F	ACCGCTTACTGGGTGTTCTACTG
sg1_R	AACCAGTAGAACACCCAGTAAGC
sg4_F	ACCGAGTGTGTTCAACTAGGTAT
sg4_R	AACATACCTAGTTGAACACACTC
sg5_F	ACCACGACACCAAATCTACAACT

sg5_R	AACAGTTGTAGATTTGGTGTCGT
sg6_F	ACCCCTCTTATTTATTACTCAGA
sg6_R	AACTCTGAGTAATAAATAAGAGG

Table S6. Primers used for vector genome quantification.

Primer	Orientation	Sequence (5'-3')
F1	Forward	CCGGAGTGTGTTCAACTAGGTATG
F2	Forward	CCGACGACACCAAATCTACAACTG
R	Reverse	CGGGCCATTTACCGTAAGTTATGTAAC

Table S7. Primers used to quantify deletion efficiency.

Primers	Orientation	Sequence (5'-3')
ΔP1	Forward	CAACAGAAACTCCCGGAACTCA
P2	Reverse	TAGATGACAGTCTGCGATTAACCAC
GAPDH_F	Forward	ATCACCATCTTCCAGGAGC
GAPDH_R	Reverse	CCTTCTCCATGGTGGTGAAG

Table S8. Primers used for amplicon sequencing.

Primer	Orientation	Sequence (5'-3')
sg6/4_F	Forward	TTGTATTTTTCGAGTCCTGTGTAAGTTTTGAAAGC
sg6/4_R	Reverse	TATTTAGATTTATCAGAATATAGATCATCTTATAGTTAGCTTTCATCACATACATTCAGA
sg1/5_F	Forward	GTTCTGAAAGATGCCCTTATTTTCCCATG
sg1/5_R	Reverse	ATACGGTATGTAACAGATTAGATGACAGTCTGC

Supplemental Figures

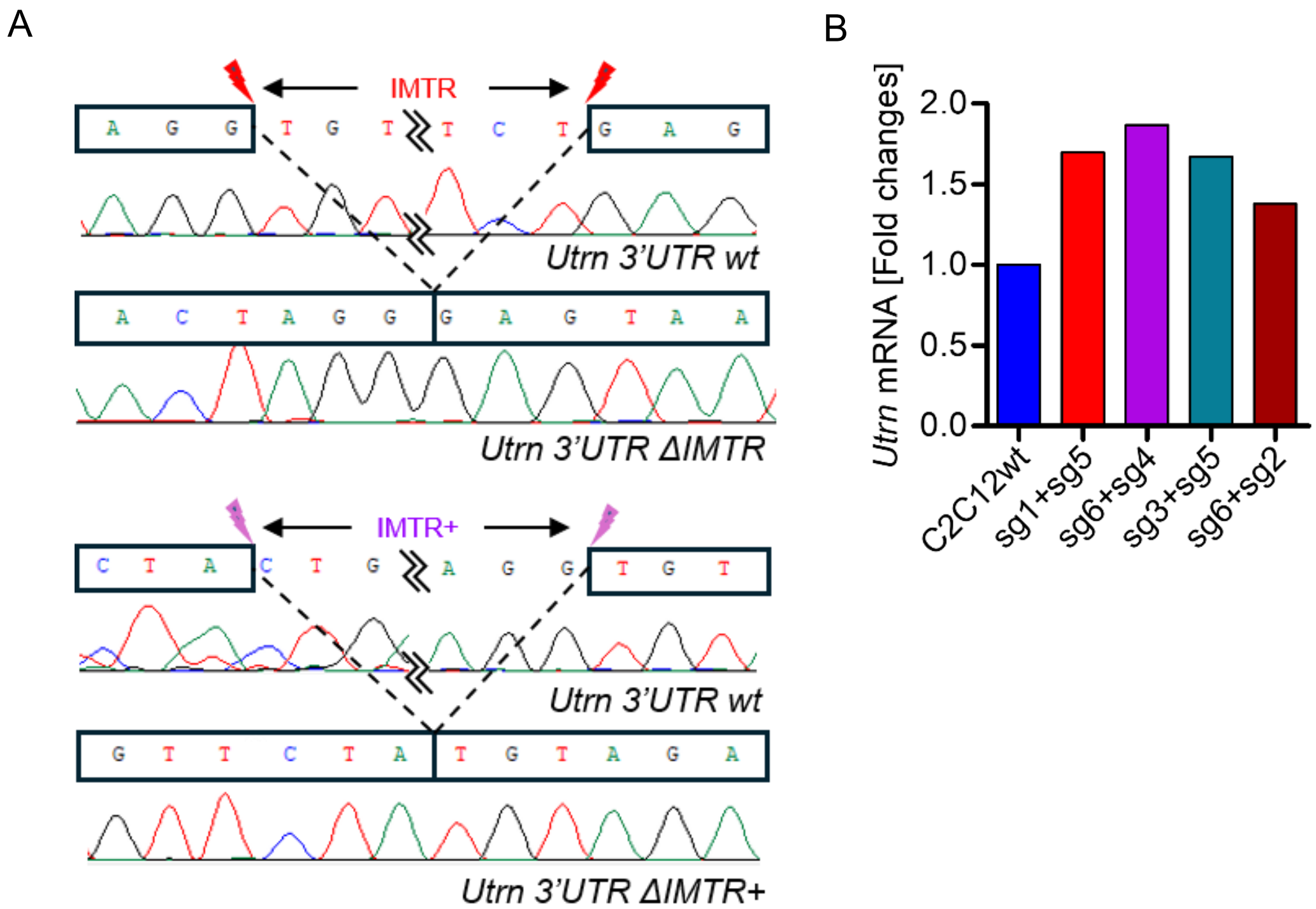

Figure S1. ***In vitro*** **utrophin gene editing increases** ***Utrn*** **mRNA expression in** ***C2C12*** **muscle cell line.**

A. Illustrative chromatograms of DNA sequence from PCR amplicons of unedited wt, ΔIMTR and ΔIMTR+ edited C2C12 muscle cell clonal lines, confirming appropriate deletion relative to the unedited DNA. The red and purple bolts show the position of the Cas9 editing sites.

B. TaqMan RT–qPCR analysis of *Utrn* mRNA levels in C2C12 wt and clonal *Utrn* Δ3′UTR cell lines (sg1+sg5, sg6+sg4, sg3+sg5 and sg6+sg2). All clonal *Utrn* Δ3′UTR cell lines showed increased expression compared with C2C12 wt cell line (n=3, technical replicates). Expression was normalized to *Gapdh*.

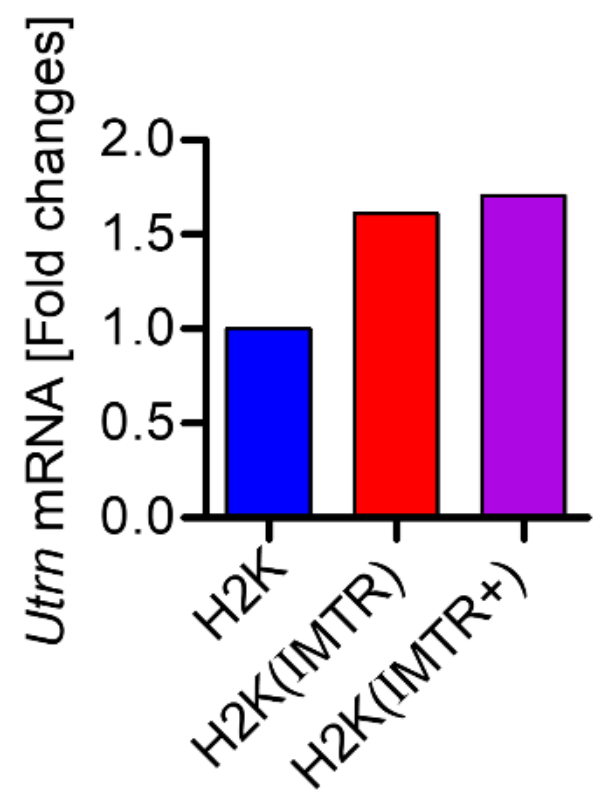

Figure S2. *In vitro* utrophin gene editing increases *Utrn* mRNA expression in MyoAAV2A-ΔIMTR/ΔIMTR⁺ transduced differentiated H2K cells. TaqMan RT–qPCR analysis of *Utrn* mRNA levels in untreated, MyoAAV2A-ΔIMTR, and MyoAAV2A-ΔIMTR⁺ transduced differentiated H2K cells. Both MyoAAV2A-ΔIMTR, and MyoAAV2A- ΔIMTR⁺ transduced H2K cells showed increased expression compared with untreated (n = 3, technical replicates). Expression was normalized to *Gapdh*.

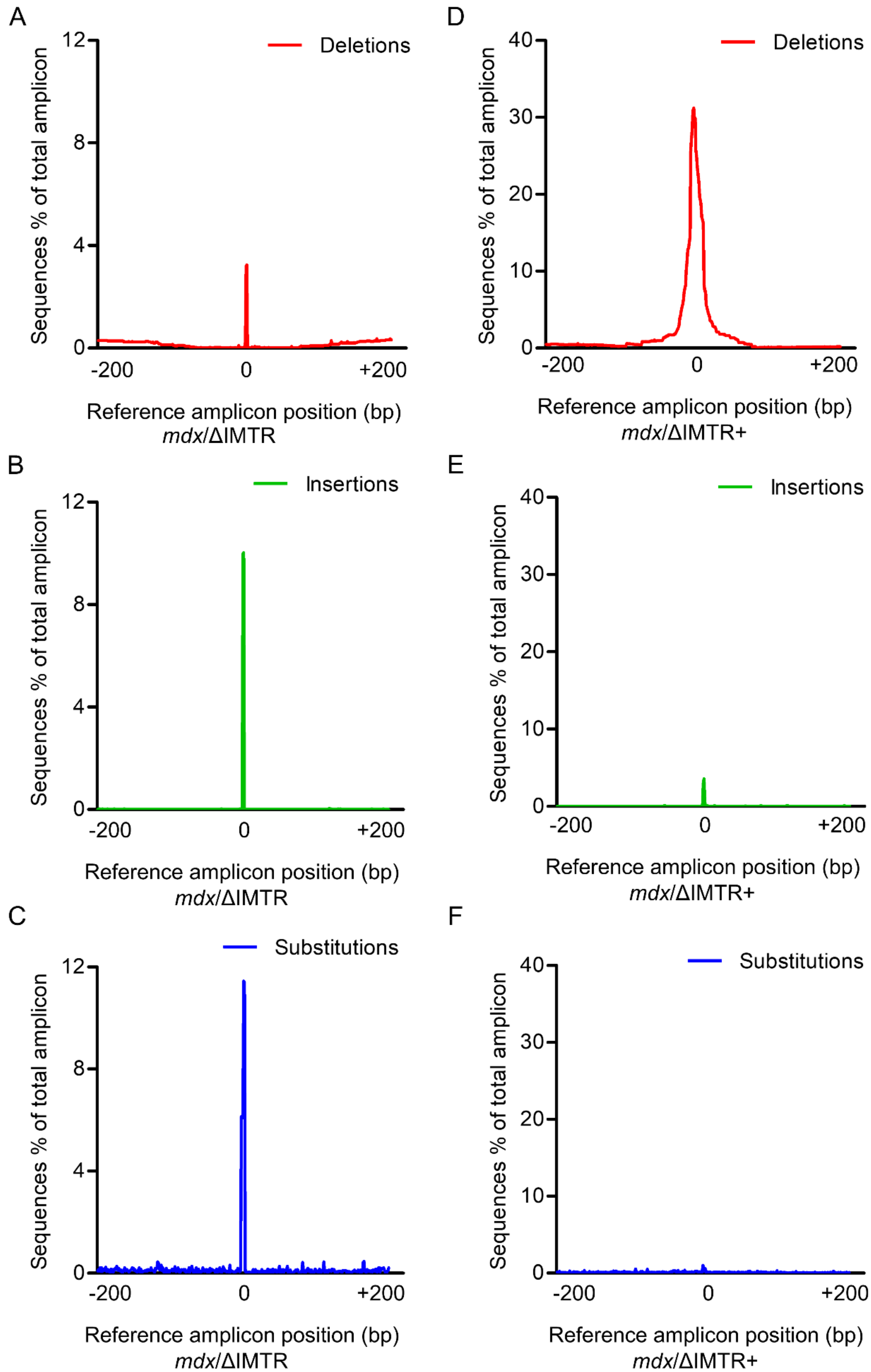
A
Deletions
Sequences % of total amplicon
12
8
4
0
-200
0
+200
Reference amplicon position (bp)
mdx/ΔIMTR
D
Deletions
Sequences % of total amplicon
40
30
20
10
0
-200
0
+200
Reference amplicon position (bp)
mdx/ΔIMTR+
B
Insertions
Sequences % of total amplicon
12
8
4
0
-200
0
+200
Reference amplicon position (bp)
mdx/ΔIMTR
E
Insertions
Sequences % of total amplicon
40
30
20
10
0
-200
0
+200
Reference amplicon position (bp)
mdx/ΔIMTR+
C
Substitutions
Sequences % of total amplicon
12
8
4
0
-200
0
+200
Reference amplicon position (bp)
mdx/ΔIMTR
F
Substitutions
Sequences % of total amplicon
40
30
20
10
0
-200
0
+200
Reference amplicon position (bp)
mdx/ΔIMTR+

G

Group		% Total mutation	bp from center
mdx/ΔIMTR	M5	57.3	±100
mdx/ΔIMTR	M6	49.8	±100
mdx/ΔIMTR	M7	53.9	±100
mdx/ΔIMTR	M8	56.5	±100
mdx/ΔIMTR$^+$	M9	91.8	±100
mdx/ΔIMTR$^+$	M10	92.8	±100
mdx/ΔIMTR$^+$	M11	92.5	±100
mdx/ΔIMTR$^+$	M12	92.7	±100

H

Editing outcome	mdx/ΔIMTR Mean ± SEM	mdx/ΔIMTR+ Mean ± SEM
Precise cut	46.05 ± 0.53	37.52 ± 0.23
Deletion	6.79 ± 0.11	31.04 ± 0.12
Insertion	8.45 ± 0.97	2.25 ± 0.03
Substitution	29.31 ± 1.03	14.10 ± 0.10
Deletion + Insertion	1.02 ± 0.09	0.21 ± 0.01
Deletion + Substitution	4.70 ± 0.25	13.42 ± 0.15
Insertion + Substitution	3.13 ± 0.19	1.10 ± 0.03
Deletion + Insertion + Substitution	0.56 ± 0.11	0.35 ± 0.02

Figure S3. Distribution and classification of sequence modifications following *in vivo Utrn* 3′UTR editing in *mdx* mice.

A–C. Representative position-specific frequencies of deletions (Red), insertions (Green), and substitutions (Blue) across the reference amplicon in the *mdx*/ΔIMTR group, generated from CRISPResso2 analysis using the expected precise deletion amplicon as the reference sequence. Mutation frequencies are plotted as the percentage of total aligned reads at each nucleotide position.

D–F. Representative position-specific frequencies of deletions (Red), insertions (Green), and substitutions (Blue) in the *mdx*/ΔIMTR$^+$ group. generated from CRISPResso2 analysis using the expected precise deletion amplicon as the reference sequence. Mutation frequencies are plotted as the percentage of total aligned reads at each nucleotide position.

G. Quantitation of position-specific frequencies of mutations. Table shows the percentage of mutations that occurred +/- 100 bp from the center of the amplicon in *mdx*/ΔIMTR and *mdx*/ΔIMTR$^+$ edited mice.

H. CRISPResso2 analysis of amplicon deep sequencing data. Data regarding frequencies of precise cuts, deletions, insertions, substitutions, deletion plus insertion, deletion plus substitution, insertion plus substitution, combined deletion, insertion, and substitution events in *mdx/*ΔIMTR and *mdx*/ΔIMTR$^+$ mice. Data are presented as mean ± SEM (n = 4 mice per group).

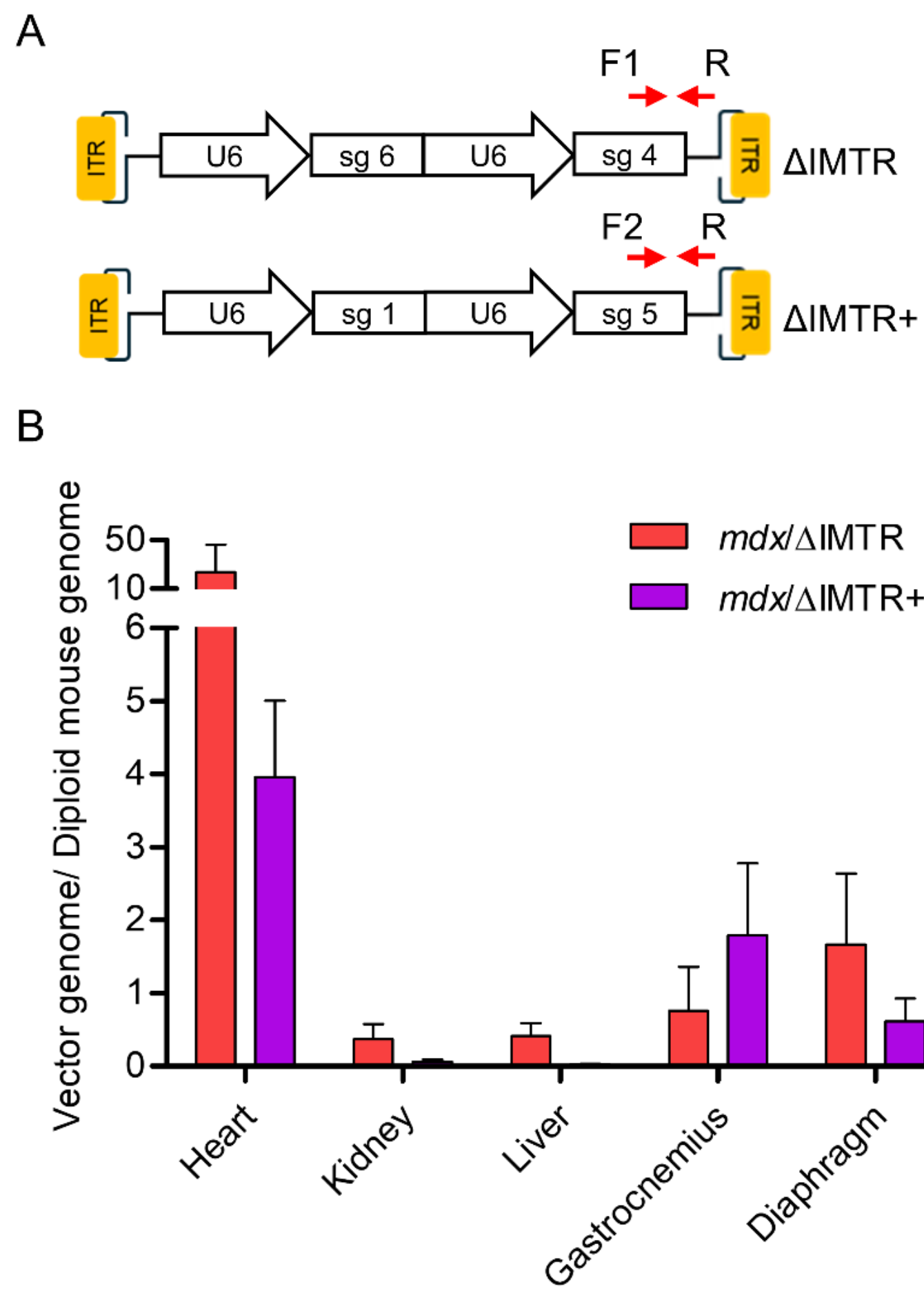

Figure S4. MyoAAV2A vector genome copy number in tissues following systemic Cas9/sgRNA delivery.

A. Schematic representation of the MyoAAV2A vector constructs used for *in vivo* genome editing, indicating primer binding sites for qPCR analysis. Primer pairs F1/R and F2/R were designed to specifically amplify the *mdx*/ΔIMTR and *mdx*/ΔIMTR^{+} vector constructs delivering the sg6/sg4 and sg1/sg5 RNAs, respectively.

B. qPCR–based quantification of MyoAAV2A vector genome copy number of the sgRNA delivery vector, in indicated tissues (heart, kidney, liver, gastrocnemius, and diaphragm) harvested 4 weeks after intraperitoneal (IP) injection. Data are presented as mean ± SEM (n = 4 mice per group).

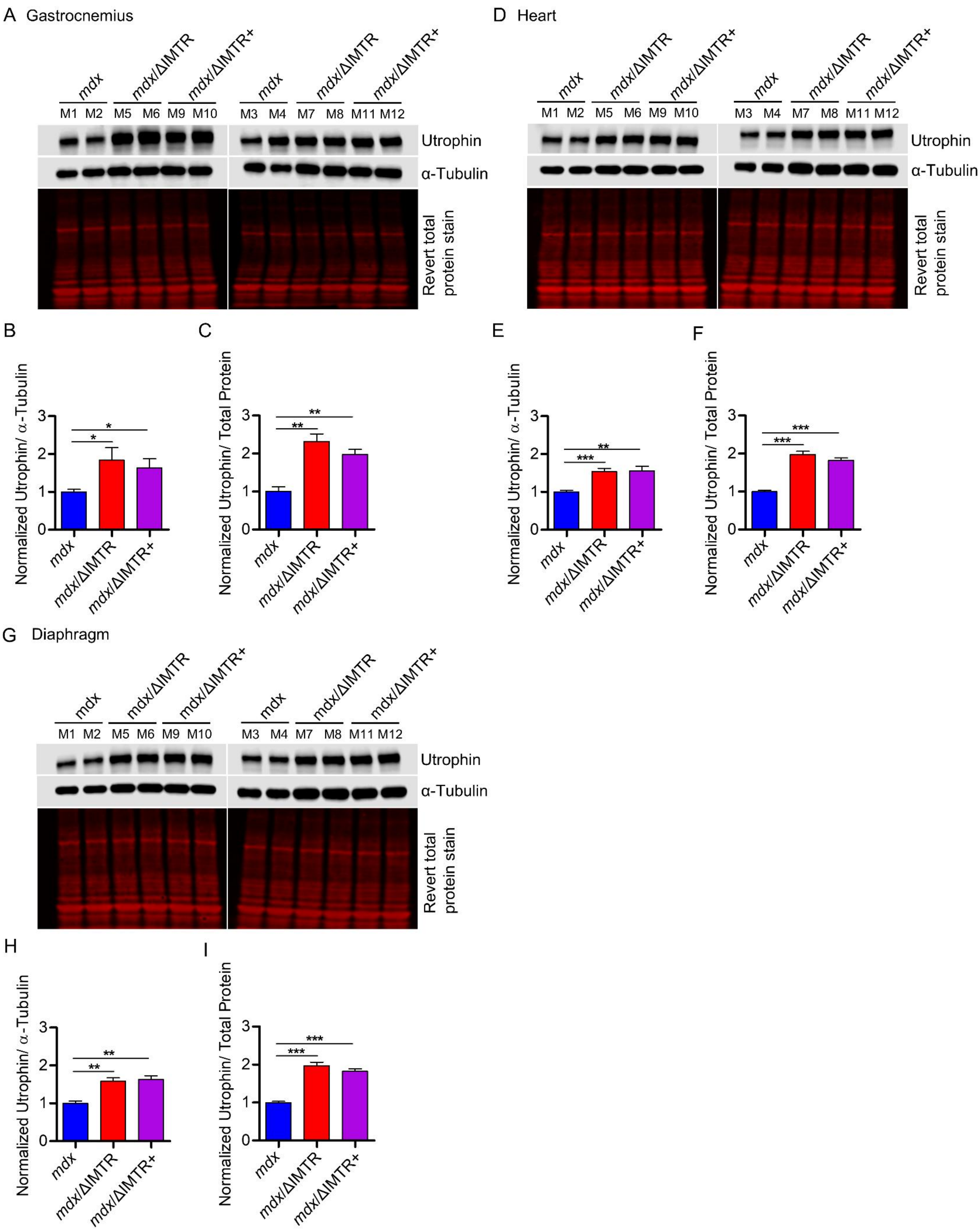
A Gastrocnemius
mdx
mdx/ΔIMTR
mdx/ΔIMTR+
M1 M2 M5 M6 M9 M10
M3 M4 M7 M8 M11 M12
Utrophin
α-Tubulin
Revert total protein stain
D Heart
B
C
E
F
Normalized Utrophin/ α-Tubulin
Normalized Utrophin/ Total Protein
0
1
2
3
*
**
***
G Diaphragm
H
I

Figure S5. *In vivo* utrophin gene editing increases utrophin expression in gastrocnemius, heart and diaphragm of *mdx* mice.

A. Representative Western blot showing utrophin protein expression in gastrocnemius muscle lysates from *mdx*, *mdx*/ΔIMTR, and *mdx*/ΔIMTR$^+$ mice. α-tubulin and total protein staining (Revert™700) were used for normalization controls.

B–C. Quantification of utrophin protein expression in gastrocnemius muscle normalized to α-tubulin **(B)** or total protein **(C)**. Data are presented as mean ± SEM (n = 4 mice per group). Relative to *mdx* controls, utrophin levels increased by ~84% and ~64% in muscle with α-tubulin normalization, and by ~132% and ~98% in muscle with total protein normalization, in the *mdx*/ΔIMTR and *mdx*/ΔIMTR$^+$ groups, respectively.

D. Representative Western blot showing utrophin protein expression in heart lysates from mdx, *mdx*/ΔIMTR, and *mdx*/ΔIMTR$^+$ mice. α-tubulin and total protein staining (Revert™700) were used as loading controls.

E–F. Quantification of utrophin protein expression in heart tissue normalized to α-tubulin **(E)** or total protein **(F)**. Data are presented as mean ± SEM (n = 4 mice per group). Relative to *mdx* controls, utrophin levels increased by ~54% and ~56% with α-tubulin normalization, and by ~97% and ~82% with total protein normalization, in the *mdx*/ΔIMTR and *mdx*/ΔIMTR$^+$ groups, respectively.

G. Representative Western blot showing utrophin protein expression in diaphragm lysates from *mdx*, *mdx*/ΔIMTR, and *mdx*/ΔIMTR$^+$ mice. α-tubulin and total protein staining (Revert™700) were used as loading controls.

H–I. Quantification of utrophin protein expression in diaphragm tissue normalized to α-tubulin **(H)** or total protein **(I)**. Data are presented as mean ± SEM (n = 4 mice per group). Relative to *mdx* controls, utrophin levels increased by ~58% and ~63% with α-tubulin normalization, and by ~95% and ~89% with total protein normalization, in the *mdx*/ΔIMTR and *mdx*/ΔIMTR$^+$ groups, respectively.

Statistical significance was determined using a two-tailed Student's *t*-test. P values are indicated as follows: $^*P < 0.05$, $^{**}P < 0.01$, and $^{***}P < 0.001$.

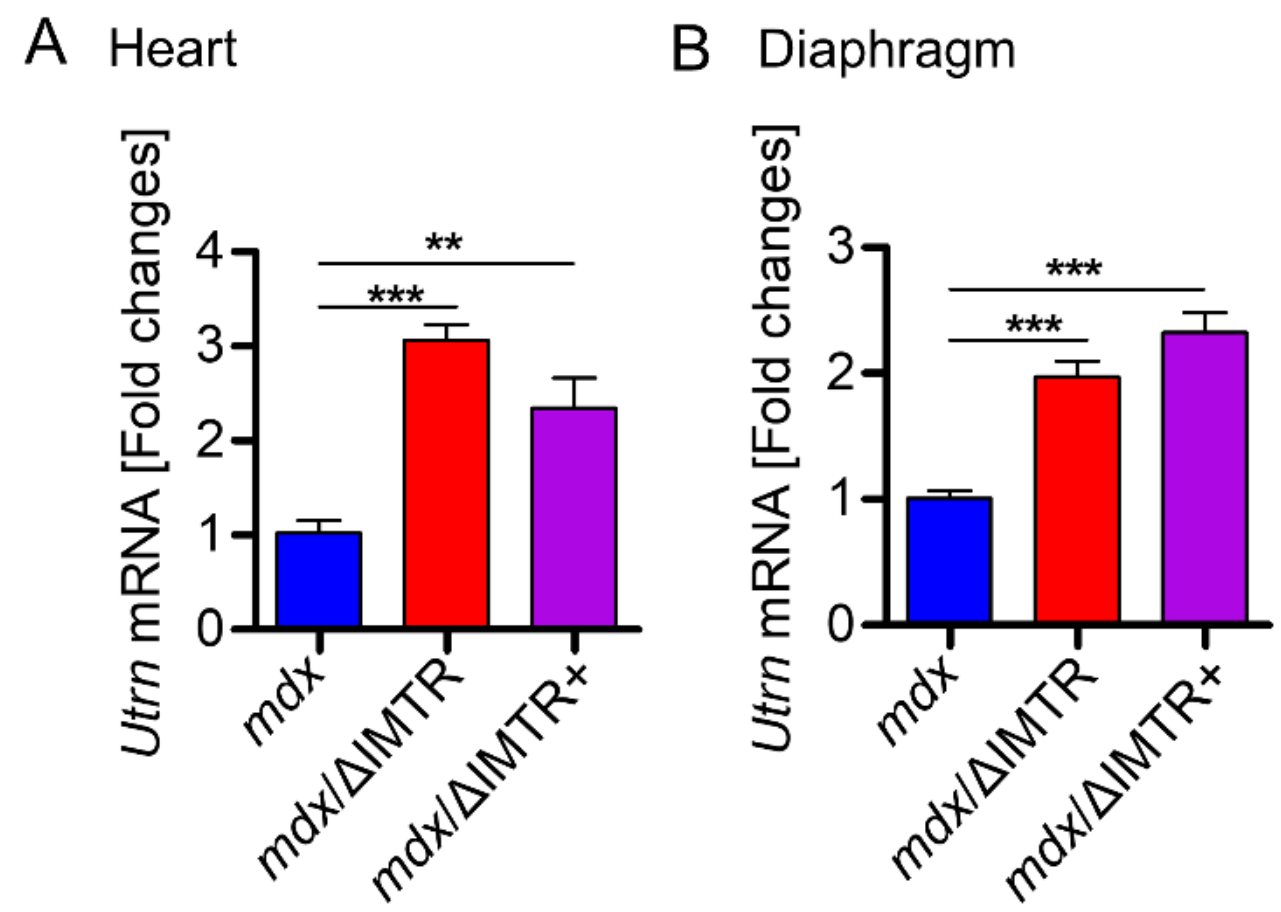

Figure S6. *In vivo* utrophin gene editing increases *Utrn* mRNA expression in *mdx* cardiac and diaphragm muscle.

A-B. TaqMan RT–qPCR analysis of *Utrn* mRNA levels in heart and diaphragm. Both *mdx*/ΔIMTR and *mdx*/ΔIMTR$^+$ groups showed increased expression compared with PBS-treated *mdx* controls. Expression was normalized to *Gapdh*. Data are presented as mean ± SEM (n = 4 mice per group). Statistical significance was determined using a two-tailed Student's *t*-test (**P < 0.01, ***P < 0.001).

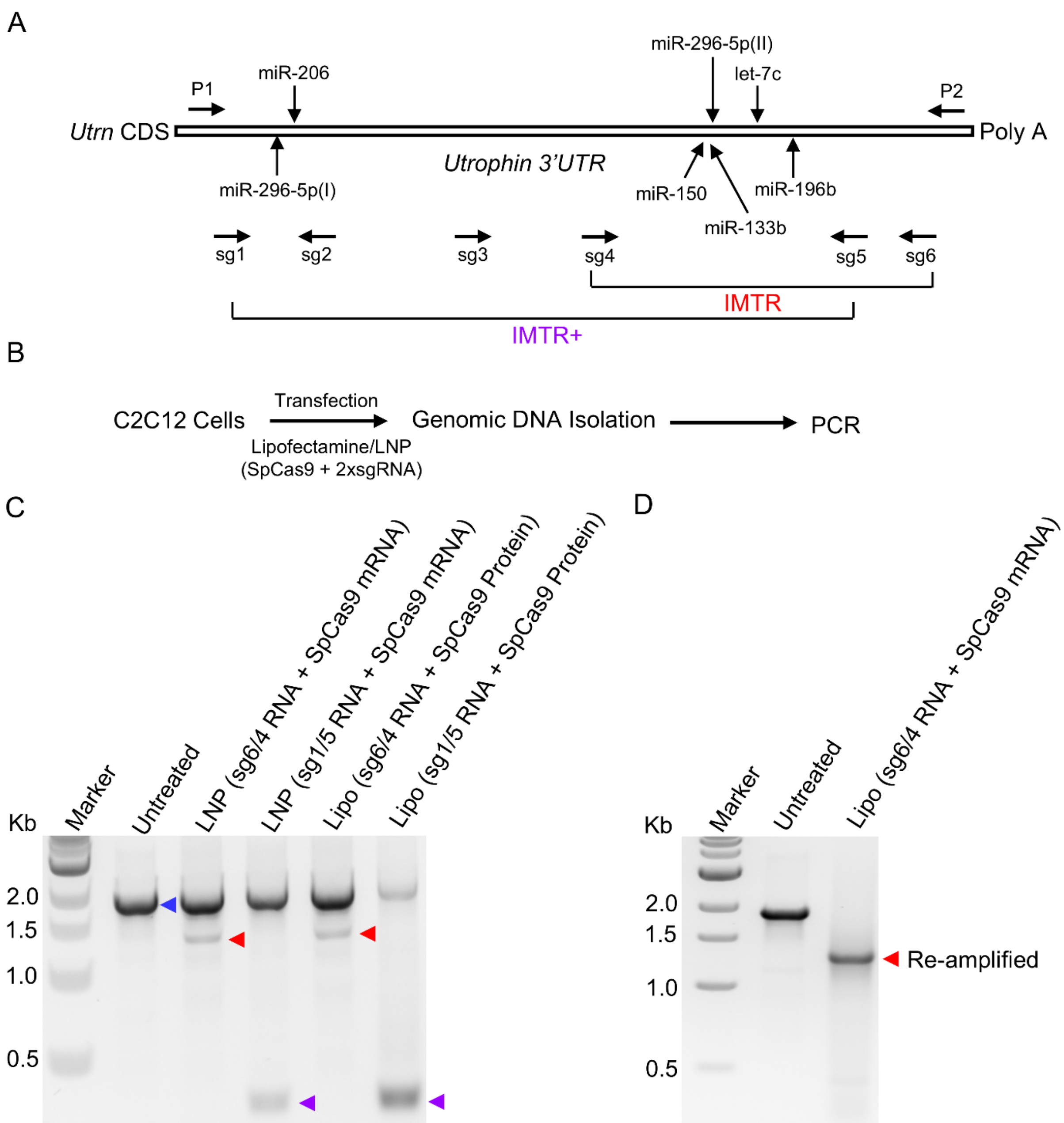

Figure S7. LNP-mediated delivery and editing of *Utrn* 3′UTR IMTR and IMTR⁺ in the C2C12 muscle cell line.

A. Schematic representation of the *Utrn* 3′UTR illustrating the relative positions of predicted microRNA target sites and the designed sgRNAs (sg1–sg6). sgRNA orientations are indicated by black arrows. Primer pairs (P1 and P2, red arrows) were used to amplify the targeted *Utrn* 3′UTR region.

B. Experimental workflow depicting C2C12 cell transfection using either LNP–mediated delivery of sgRNA pairs with SpCas9 mRNA or Lipofectamine-mediated delivery of sgRNA pairs with SpCas9 mRNA or protein, followed by genomic DNA isolation and PCR-based deletion screening.

C-D. Agarose gel electrophoresis of genomic PCR products from C2C12 cells transfected with different sgRNA combinations. The unedited *Utrn* 3′UTR yields an approximately 1.9 kb amplicon (blue arrow), whereas successful genome editing generates shorter deletion products corresponding to ΔIMTR (red arrows) and $\Delta IMTR^{+}$ (purple arrows).

References

1. Karikó, K., Muramatsu, H., Welsh, F.A., Ludwig, J., Kato, H., Akira, S., and Weissman, D. (2008). Incorporation of Pseudouridine Into mRNA Yields Superior Nonimmunogenic Vector With Increased Translational Capacity and Biological Stability. Mol. Ther. *16*, 1833–1840. https://doi.org/10.1038/mt.2008.200.

2. Karikó, K., Muramatsu, H., Keller, J.M., and Weissman, D. (2012). Increased Erythropoiesis in Mice Injected With Submicrogram Quantities of Pseudouridine-containing mRNA Encoding Erythropoietin. Mol. Ther. *20*, 948–953. https://doi.org/10.1038/mt.2012.7.

3. Andries, O., Mc Cafferty, S., De Smedt, S.C., Weiss, R., Sanders, N.N., and Kitada, T. (2015). N1-methylpseudouridine-incorporated mRNA outperforms pseudouridine-incorporated mRNA by providing enhanced protein expression and reduced immunogenicity in mammalian cell lines and mice. J. Controlled Release *217*, 337–344. https://doi.org/10.1016/j.jconrel.2015.08.051.

4. Baiersdörfer, M., Boros, G., Muramatsu, H., Mahiny, A., Vlatkovic, I., Sahin, U., and Karikó, K. (2019). A Facile Method for the Removal of dsRNA Contaminant from In Vitro-Transcribed mRNA. Mol. Ther. - Nucleic Acids *15*, 26–35. https://doi.org/10.1016/j.omtn.2019.02.018.
